# Bringing order to protein disorder through comparative genomics and genetic interactions

**DOI:** 10.1186/gb-2011-12-2-r14

**Published:** 2011-02-16

**Authors:** Jeremy Bellay, Sangjo Han, Magali Michaut, TaeHyung Kim, Michael Costanzo, Brenda J Andrews, Charles Boone, Gary D Bader, Chad L Myers, Philip M Kim

**Affiliations:** 1 Department of Computer Science and Engineering, University of Minnesota, 200 Union Street SE, Minneapolis, MN 55455, USA; 2 The Donnelly Centre, University of Toronto, 160 College Street, Toronto, ON M5S 3E1, Canada; 3 Banting and Best Department of Medical Research, University of Toronto, 160 College Street, Toronto, ON M5S 3E1, Canada; 4 Department of Molecular Genetics, University of Toronto, 160 College Street, Toronto, ON M5S 3E1, Canada; 5 Department of Computer Science, University of Toronto, 160 College Street, Toronto, ON M5S 3E1, Canada

## Abstract

**Background:**

Intrinsically disordered regions are widespread, especially in proteomes of higher eukaryotes. Recently, protein disorder has been associated with a wide variety of cellular processes and has been implicated in several human diseases. Despite its apparent functional importance, the sheer range of different roles played by protein disorder often makes its exact contribution difficult to interpret.

**Results:**

We attempt to better understand the different roles of disorder using a novel analysis that leverages both comparative genomics and genetic interactions. Strikingly, we find that disorder can be partitioned into three biologically distinct phenomena: regions where disorder is conserved but with quickly evolving amino acid sequences (flexible disorder); regions of conserved disorder with also highly conserved amino acid sequences (constrained disorder); and, lastly, non-conserved disorder. Flexible disorder bears many of the characteristics commonly attributed to disorder and is associated with signaling pathways and multi-functionality. Conversely, constrained disorder has markedly different functional attributes and is involved in RNA binding and protein chaperones. Finally, non-conserved disorder lacks clear functional hallmarks based on our analysis.

**Conclusions:**

Our new perspective on protein disorder clarifies a variety of previous results by putting them into a systematic framework. Moreover, the clear and distinct functional association of flexible and constrained disorder will allow for new approaches and more specific algorithms for disorder detection in a functional context. Finally, in flexible disordered regions, we demonstrate clear evolutionary selection of protein disorder with little selection on primary structure, which has important implications for sequence-based studies of protein structure and evolution.

## Background

Many proteins include extended regions that do not fold into a native fixed conformation. These are referred to as being intrinsically unstructured or disordered. A possible utility of such regions was first suggested over 70 years ago by Linus Pauling, who speculated that their flexibility aids in antibody creation [1]. Recent advances in computational prediction of disordered regions in amino acid sequences have greatly expanded our awareness of the widespread occurrence of disordered regions and the number of proteins whose structure is dominated by such regions (intrinsically disordered proteins or IDPs). Interestingly, protein disorder is more prevalent in complex organisms, accounting for 33% of the residues in the human proteome, but only a few percent of residues in *Escherichia coli*, suggesting it may play a major role in the evolution of complexity [2].

Protein disorder is a diverse and complex phenomenon. On a biophysical level, there exists a continuum of structure and disorder in the proteome. At one extreme, there are proteins that are almost entirely unstructured and natively form a coil; some may fold upon binding a ligand, and thereby undergoing a disorder to structure transition. Other proteins that are structurally more constrained, but still considered disordered, adopt a molten globule conformation [3]. Highly structured proteins, which conform to the classical model of protein structure, occupy the other extreme on this spectrum, but even they often possess locally disordered regions [3]. On a functional level, there are numerous and varied roles with which IDPs have been associated, including signaling, cellular regulation, nuclear localization, chaperone activity, RNA and DNA binding, protein binding and dosage sensitivity [4,5], antibody creation [6], and splicing [7]. Also, IDPs have been implicated in a variety of diseases, including cancer [8], and neurodegenerative and cardiovascular diseases [6].

While the importance and widespread occurrence of IDPs is undisputed, a mechanistic understanding of the specific structural and functional roles of disorder is still lacking. Here, we systematically analyze and structure the different functions of disorder through the use of genetic interactions (GIs) and comparative genomics. We use two different, but related, concepts to partition disordered regions into three categories. Our analysis partitions what is currently only generally characterized as 'disorder' into several fundamentally different phenomena with distinct properties and functions.

## Results

### Genetic interaction hubs tend to have more disordered residues

**Figure 1:**
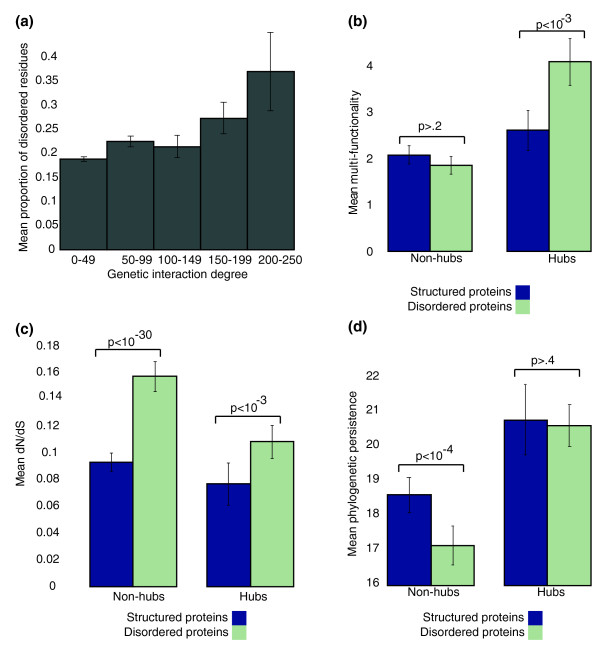
**Genetic interactions distinguish different roles of disorder**. **(a) **Percentage of disordered residues of yeast proteins by their number of GIs. **(b) **Multi-functionality (see Materials and methods) for disordered and structured GI hubs and non-hubs. Hubs are genes in the top 90th percentile (above 90 interactions) of GIs while non-hubs are in the bottom 50th percentile (below 15 interactions). **(c) **Evolutionary constraint on sequence (dN/dS ratio) on hubs and non-hubs. In both cases disordered proteins have a significantly higher dN/dS than structured proteins. **(d) **Evolutionary constraint measured by the presence of orthologs in other yeast species (phylogenetic persistence). While disordered non-hubs are less conserved than structured non-hubs, the disordered hubs are as conserved as structured hubs. *P*-values were computed with a Wilcoxon test, and error bars represent boot-strapped 95% confidence intervals.

Despite the apparent importance of disorder in mediating important protein functions [4], our knowledge is still limited in terms of its specific functional roles. The yeast GI network offers a new opportunity for global insights into the role of disorder in protein function [9]. Briefly, GIs are defined as pairs of genes whose combined mutation or deletion leads to an unexpected double mutant phenotype. Here we limit our attention to negative interactions; these are interactions in which the double mutant is significantly less fit than would be predicted by the fitnesses of the single mutants. Interestingly, it has been observed that the number of GIs of a gene (GI degree) is correlated with the percentage of disordered regions in the gene product [9] (Figure 1a). GI degree is also correlated with different measures of multi-functionality (number of gene ontology (GO) annotations, phenotypic capacitance [10] and chemical-genetic sensitivity [11]), suggesting that the presence of disordered regions may underlie the highly pleiotropic roles of some proteins.

The relationship between disorder and multi-functionality appears to depend on whether a gene is a hub in the GI network (that is, the gene is associated with a large number of GIs). Specifically, within the set of the GI hubs (> 90 percentile in GI degree), disorder of the gene product is a strong predictor of multi-functionality (r = 0.22, *P *< 10^-12^; Figure 1b), suggesting it is able to distinguish highly functionally versatile GI hubs from genes with more limited functional roles that simply exhibit a large number of GIs. However, this trend is absent on the set of non-GI hubs (< 50 percentile in GI degree) where there is no significant correlation between the amount of disorder and the number of annotated functions (r = -0.02, *P *> 0.3). This stark difference suggests that disorder plays a highly functional role on the set of proteins that have many GIs while disorder outside these genes is either less functional or simply of a markedly different nature. A similar distinction can be observed for protein-protein interactions: disorder is significantly correlated with protein-protein interaction degree on GI hubs (r = 0.16, *P *< 3 × 10^-3^; Figure S1 in Additional file 1) while no such correlation holds on non-GI hubs (r = -0.01, *P *> 0.5). Thus, the GI network appears to provide a clear means of defining a set of proteins where the disorder plays a key functional role.

Despite their seeming functional importance, disordered regions of proteins have previously been associated with swiftly evolving, less conserved sequences, presumably because of lower structural constraint [12]. We were intrigued by this property because, in general, GI hubs exhibit significantly lower rates of evolution (for example, measured by the dN/dS ratio) and tend to be conserved more broadly across species [9]. Indeed, we found that even among GI hubs, disordered proteins have significantly elevated rates of evolution. This trend is consistent outside the hubs as well (Figure 1c). However, disordered GI hubs are just as conserved phylogenetically as measured by their appearance across the yeast clade (Figure 1d). Thus, while the amino acid sequences tend to evolve faster for disordered GI hubs, they appear to be as phylogenetically constrained at the gene level as other GI hubs. Interestingly, outside of GI hubs, this is not true: non-GI hubs that are disordered tend to be less conserved across the yeast clade compared to their structured counterparts (Figure 1d). These observations relating disordered proteins to the GI network raise an interesting paradox. While the presence of disordered regions appears to be directly connected to their importance in the genetic network, there appears to be little evolutionary sequence constraint on these regions.

### Many disordered residues are conserved across species

**Figure 2:**
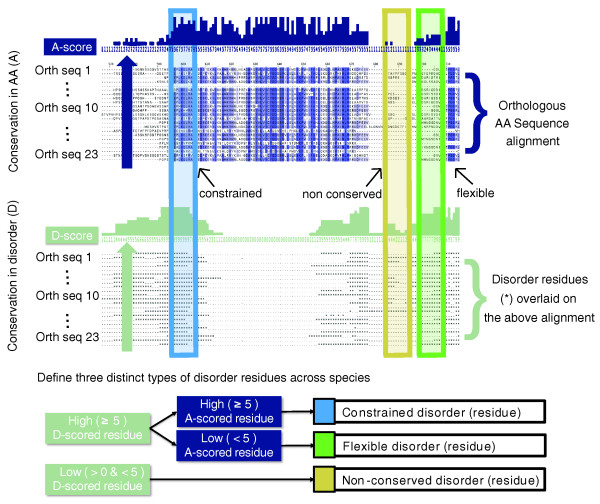
**Two forms of conservation on disorder**. Schematic of computing disorder conservation and amino acid (AA) sequence conservation. After alignment, the percentage of sequences in which a residue is disordered is computed. Similarly, we compute the percentage of sequences in which the amino acid itself is conserved. A residue is considered to be conserved disorder if the property of disorder is conserved in ≥ 50% of species and sequentially conserved if the amino acid is conserved in ≥ 50% of species. Disordered residues in which both sequence and disorder are conserved are referred to as *constrained disorder*. Disordered residues in which disorder is conserved but not the amino acid sequence are referred to as *flexible disorder*. Residues which are disordered in *S. Cerevisiae *but not cases of conserved disorder are referred to as *non-conserved disorder*.

The counter-intuitive evolutionary pressure on disordered proteins motivated us to undertake a comparative analysis of disordered regions across the yeast clade. We hypothesized that functionally important disordered regions, such as those present in GI hubs, would be conserved as disorder across species (that is, also disordered, even if the underlying amino acid sequence was different) independent of rate of evolution. We therefore assessed the conservation of disorder on the residue level, which was also recently addressed by Chen *et al*. [13,14]. Specifically, we predicted which residues were disordered for all *Saccharomyces cerevisiae *genes and their orthologs in the 23 species of the yeast clade using DISOPRED2 [2], an algorithm that has been shown to predict disordered regions reliably [15]. For each disordered residue, we defined a measure of conserved disorder as the percentage of orthologs in which that residue is disordered as well (Figure 2). We operationally define conserved disordered residues as those with greater than 50% of disorder conservation.

Consistent with the general observations by Chen and co-workers [13,14], we found that there is a surprisingly high rate of conservation of disordered regions: over 50% of disordered regions are conserved through 90% of the orthologs considered. Notably, disorder is conserved in many regions even where the specific amino acids are not conserved in the same regions, which explains the elevated dN/dS that has been previously associated with disorder [12] (Figure 2). However, consistent with the stability of disorder across the yeast clade, we find that changes of amino acids in disordered regions are biased towards hydrophilic residues associated with disordered regions and away from hydrophobic residues (Figure S2 in Additional file 1). This result suggests that, despite a high evolutionary rate at the sequence level, there is substantial evolutionary pressure to keep these regions disordered.

### Disorder can be systematically classified

**Figure 3:**
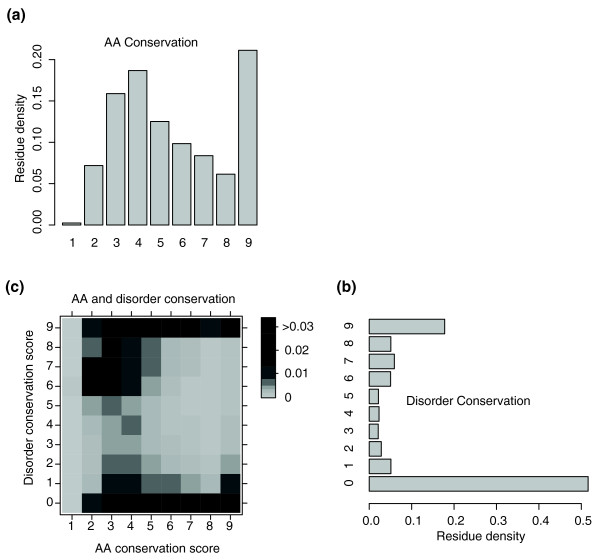
**Densities of disorder- and amino acid-conserved residues by their scores**. Densities of disorder and amino acid conservation scores across all alignments of approximately 5,000 orthologous groups from 23 yeast species. **(a) **Histogram of the amino acid (AA) conservation scores. **(b) **Histogram of disorder conservation scores. **(c) **Two-dimensional histogram of both amino acid and disorder conservation scores.

Regions in which disorder is highly conserved across the yeast clade exhibit a wide range of amino acid conservation rates (Figure 3). We reasoned that the degree of constraint on the precise underlying sequence (as opposed to the more general property of disorder) might highlight distinct subclasses of functional disorder. To test this hypothesis, we divided conserved disordered regions into those where the underlying amino acid sequence is also conserved ('constrained disorder'), and the regions where there appears to be selection on the structural property of disorder itself rather than the specific sequence ('flexible disorder'; Materials and methods; Figure 2). Disordered residues that were not conserved across the yeast clade were considered as a separate, third class ('non-conserved disorder'; Figure S3 in Additional file 1). It is important to note that these results do not depend on the disorder predictor algorithm and core results were qualitatively replicated using DisEMBL [16] instead of DISOPRED2 (Figure S4 in Additional file 1). Furthermore, the three classes also appear to be robust to various perturbations of the particular parameter choices of the method (Figures S5, S6, S7, and S8 in Additional file 1). In addition, flexible disorder was more robust to random simulated mutations (Figure S9 in Additional file 1), which is notable given the general fragility of disorder to mutation reported by [17].

**Figure 4:**
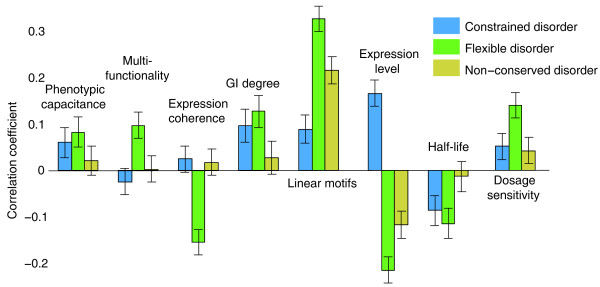
**Properties associated with types of disorder**. Correlation coefficients of different genomic features with percent constrained disorder, percent flexible disorder and percent non-conserved disorder. Error bars represent 95% confidence intervals.

The three classes of disorder exhibit widely different properties (Figure 2b). First, while disorder is generally thought to be important in proteins with regulatory and signaling functions, we find that this is true only for flexible disorder. For instance, proteins enriched in flexible disorder have high phenotypic capacitance and are multifunctional. Moreover, they exhibit low-expression coherence, that is, are connectors in the cellular network, consistent with a regulatory role [18]. Finally, flexible disorder is highly correlated with occurrence of linear motifs and GI degree, also consistent with signaling or regulatory roles. The respective associations for all the above properties with either constrained or non-conserved disorder are much weaker and, in most cases, not significant, suggesting that the regulatory properties of disorder are best captured by flexible disorder. Secondly, disordered proteins have recently been found to be expressed at a low level and have tightly controlled expression [4]. We find this only true for proteins enriched in flexible disorder: flexible disorder is negatively correlated with gene expression level, while constrained disorder shows either a positive or no correlation depending on the inclusion of ribosomal proteins (Figure 4; Figure S7 in Additional file 1). Also, while genes enriched in non-conserved disorder appear to be expressed at a low level, there appears no evidence for tighter expression control as measured by half-life. Thirdly, a recent study found disordered proteins to exhibit high dosage sensitivity [5]. We again find that this is a hallmark of flexible disorder (Figure 4), whereas constrained disorder is only weakly associated with this property. Non-conserved disorder shows little or much weaker association with most of these features, suggesting that the functional hallmarks of this class are less obvious. Indeed, we find that proteins enriched for non-conserved disorder have less confident disorder as scored by DISOPRED2 (Figure S10 in Additional file 1). However, our inability to identify functional roles for non-conserved disorder does not preclude the possibility of its functionality.

**Figure 5:**
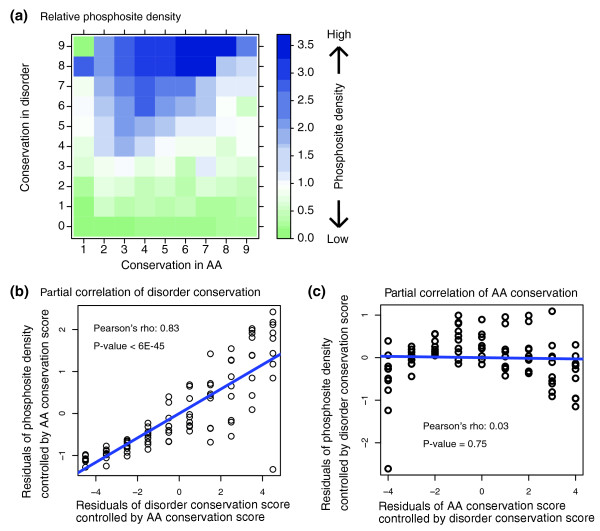
**Properties associated with types of disorder**. **(a) **Heatmap of enrichment (density over background) of phosphosites in terms of disorder and amino acid conservation. **(b) **Partial correlation of phosphosite density and disorder conservation with respect to amino acid conservation (see Materials and methods). **(c) **Partial correlation of phosphosite density and conserved amino acid sequence with respect to disorder conservation.

Because of their recognized importance for signaling pathways, we next turned our attention towards phosphosites and linear motifs. It has been noted previously that phosphosites and other recognized linear motifs often appear in disordered regions of proteins [19]. As these motifs are crucial for signaling pathways, their occurrence in these regions certainly has strong functional consequences. In a detailed analysis at the residue level, we find that disorder conservation is strongly correlated with the placement of phosphosites (Figure 5a). In particular, we find that the relative density of phosphosites increases dramatically for residues with higher disorder conservation (Figure 5b). Conversely, the correlation of phosphosite density with amino acid conservation is weak (Figure 5c). Likewise, we find similar results for linear motif placement (Figure S11 in Additional file 1). In both cases, the partial correlation with conserved disorder, when controlling for amino acid conservation, remains strong, while the partial correlation between amino acid conservation and phosphosite or linear motif density disappears when controlling for conserved disorder. Conversely, neither linear motifs nor phosphosites show enrichment in residues that exhibit non-conserved disorder, which suggests that non-conserved disorder may not be functionally relevant in this context.

Given our comparative genome-based classification of disorder, we revisited our earlier observation regarding the correlation between protein disorder and multi-functionality on GI hubs. As described earlier, we observed that within the set of the GI hubs (> 90 percentile in GI degree), disorder of the gene product is a strong predictor of multi-functionality (r = 0.22, *P *< 10^-12^; Figure 1b) while this trend does not hold on the set non-GI hubs (< 50 percentile in GI degree). Thus, we reasoned that the disorder present in GI hubs may exhibit different abundances across our classes. Indeed, we did find evidence that disordered regions tend to be significantly more conserved among GI hubs than non-hubs (*P *< 10^-6^; Figure S12 and Table S1 in Additional file 1). Furthermore, flexible disorder appears to account for the correlation between disorder and multi-functionality observed among the GI hubs since controlling for flexible disorder destroys the correlation (*P *> 0.5), while a strong correlation is maintained when controlling for the level of constrained disorder (r = 0.15, *P *< 0.01).

**Figure 6:**
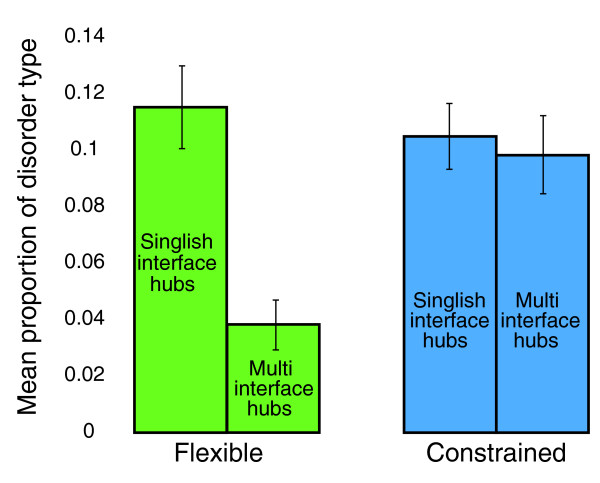
**Singlish and multi-interface hubs have different proportions of flexible and constrained disorder**. The mean proportion of flexible disorder and constrained disorder in singlish-interface and multi-interface protein interaction hubs. While both have a similar level of constrained disorder, singlish hubs are heavily enriched for flexible disorder. Error bars represent 95% confidence intervals.

Interestingly, the set of highly disordered GI hubs is also significantly enriched for protein interaction hubs that bind temporally disparate partners (singlish interface hubs as defined in [20]) when compared with disordered non-hubs or non-disordered hubs (*P *< 10^-5^; Figure S13 in Additional file 1). In fact, the distinction between flexible and constrained disorder can be used to differentiate between singlish-interface hubs and the so-called multi-interface hubs, which typically bind their partners simultaneously (as defined in [20]): singlish hubs have more flexible disorder than multi-interface hubs (*P *< 10^-13^), while there is no significant difference in terms of constrained-disorder (*P *> 0.1; Figure 6).

### Flexible and constrained disorder show different functional associations

**Figure 7:**
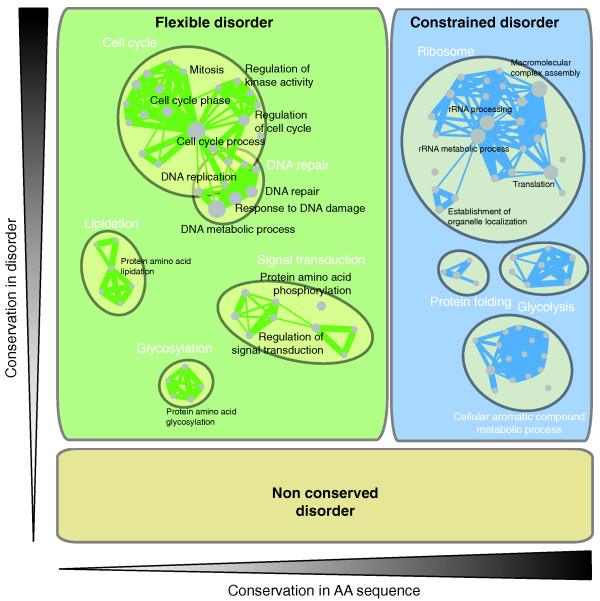
**Disorder splits into three distinct phenomena**. Functional enrichment maps of proteins enriched in flexible disorder versus constrained disorder. The area of each rectangle is proportional to the representation of that type of disorder in the alignments. Related GO terms are grouped based on gene overlap (see Materials and methods; Figures S20, S21 and S22 in Additional file 1).

The above results indicate that flexible disorder and constrained disorder are markedly different phenomena based on a variety of physiological and phenotypic data. On the one hand, flexible disorder corresponds to what we refer to as 'classic disorder': these are intrinsically unstructured regions, which evolve rapidly and present short linear motifs to signaling domains or protein kinases. Flexible disorder is thus a central player in signaling, which is confirmed by a GO enrichment analysis - all top enriched terms are related to regulation, including transcription factors, chromatin modifiers, and signaling pathways and DNA binding proteins (Figure 7; Table S2 in Additional file 2).

In contrast, proteins with a high level of constrained disorder exhibit dramatically different functional characteristics. Constrained disordered proteins are enriched in genes involved in ribosome biogenesis or function, RNA binding and protein chaperone activity (Figure 7; Table S2 in Additional file 2). Some of these functions have been previously associated with conserved disorder [14], but our analysis suggests they are even more specifically associated with regions that are under tight sequence constraint, which is not generally true of regions that have properties characteristic of 'classic' disorder.

Given the dichotomy in functions arising from the presence or lack of sequence constraint, we explored the positions of these regions with respect to predicted domains. We find that flexible disordered residues rarely reside inside structured domains, consistent with the idea that they would localize to loops to present highly flexible linear motifs to their signaling partners. Conversely, constrained disordered residues lie within domains significantly more frequently than flexible residues, though occurring well below the level of the genomic background (Figures S14 and S15 in Additional file 1). The particular domains in which constrained disorder residues are enriched confirmed the location of these regions within RNA-binding ribosomal proteins and protein chaperones (GroEL-like chaperone, ATPase, Translation protein SH3-like, AAA ATPase, core; Table S3 in Additional file 2).

The highly distinct functional and positional characteristics associated with these two classes of disorder suggest that they are very different phenomena. On the one hand, flexible disorder is closest to what is canonically understood as protein disorder, that is, these are structurally flexible, fast evolving sequences with involvement in signaling. A good example of flexible disorder is found in the serine-arginine protein kinase Sky1 (YMR216C), similar to human SRPK1, which regulates proteins involved in mRNA metabolism and cation homeostasis. The region containing residues 712-737, conserved for disorder across orthologs but not sequence, is located at the end of the kinase (Figure S16 in Additional file 1). This carboxy-terminal disordered loop interacts with the activation loop of the kinase [21] and is likely involved in the regulation of kinase activity. Likewise, the corresponding region exhibits flexible disorder in many of the related cyclin-dependent kinases [22]. For example, in Bur1, this region contains flexible disorder and also harbors multiple phosphosites and linear motifs, underlining its importance in signaling (Figure S17 in Additional file 1).

On the other hand, our results suggest that constrained disorder can often adopt fixed conformation. As has been previously suggested, some disordered proteins are likely to undergo disorder-to-order transitions upon binding of their targets [3], and we speculate this is a hallmark of the constrained disorder class. In the case of ribosomal biogenesis and RNA-binding structural proteins, they become structured upon binding RNA. This imposes a high degree of local structural constraint on them, which results in elevated constraint on the actual amino acid sequence. For instance, in Rpl5 a region of constrained disorder can be observed immediately before an alpha helix that forms the carboxy-terminal end of the amino acid sequence (Figure S18 in Additional file 1). The role of this region was specifically investigated in [23], and they report strong evidence for a disorder-to-order transition of this region upon the binding of Rpl5 to 5S rRNA. We also found an enrichment for constrained disorder among protein chaperones, where disordered regions appear to be involved in the binding of client proteins. For example, the HSP90 heat shock protein (HSC82/HSP82) contains long regions of constrained disorder (Figure S19 in Additional file 1). In particular, the constrained disordered region from 590-600 is conserved throughout the bacterial kingdom, is localized at the inner surface of the barrel-shaped protein and has been directly implicated in the chaperone activity of this protein. It has been previously speculated that this disordered region may play a role in entropy transfer and the refolding of clients through a disorder-to-order transition [24]. However, there is little direct experimental evidence about the precise role of disorder in chaperone function. We hypothesize that, in general, the tight sequence conservation of constrained disorder is required in regions that assume a structured conformation, even if this conformation is only assumed in a transient fashion as in the case of HSP90 or more permanently as in the case of Rpl5.

## Discussion

In this work, we show that protein disorder can be partitioned into three biophysically and biologically distinct phenomena. The first two, flexible and constrained disorder, capture different functional characteristics: flexible disorder appears to be strongly associated with signaling and regulation while constrained disorder is associated with chaperones and ribosomal proteins. Flexible disorder appears to be largely responsible for many of the characteristics traditionally associated with disordered regions. On the other hand, non-conserved disorder does not seem to have obvious functional hallmarks by our analysis. While we discovered these categories using a comparative genomics approach that exploits evolutionary signatures, they ultimately are likely to correspond to biophysically different phenomena. In a similar fashion, modern secondary prediction methods make use of evolutionary information in the form of sequence profiles, while they discover biophysical properties.

Several classification schemes for protein disorder have been described in previous studies, including categorizations based on structural descriptions [3,25], molecular function [26], or data-driven unsupervised partitions [27]. In particular, the functional characterization put forth in [26] (Figure S24 in Additional file 1) has an interesting overlap with the flexible and constrained categories defined here. Tompa [26] first makes a distinction between proteins whose disordered regions perform a purely mechanical function (for example, entropic chains) from those that have the capacity to bind other proteins or small molecules (recognition). A similar division is made by [25] between disordered regions that can at least transiently fold ('folders') from regions that never fold ('unfolders'). There the authors claim that entropic chains are necessarily unfolders, while recognition regions are necessarily folding regions. The yeast nucleoporin *NUP2*, a canonical example of entropic chains, appears to contain long regions of flexible disorder. In fact, 22% of its residues are cases of flexible disorder (the background rate is 9%) while only 12% is constrained disorder (the background rate is 7%). This is consistent with the fact that the role of such regions does not require strict residue conservation and it is tempting to speculate that other entropic chains are also cases of flexible disorder.

Despite some evidence that flexible disordered regions as defined here may correspond to entropic chains, the previously defined category of recognition proteins (folders) appears to contain clear cases of both flexible and constrained disorder. In particular, the subcategory of 'display sites' seems to correspond to our notion of flexible disorder, given its enrichment for linear motifs and association with signaling proteins. These appear to be cases of a relatively short recognition motif contained in a longer disordered region [28], and it has been previously observed that, while functional recognition motifs are well conserved, the surrounding disordered region may evolve quickly [29]. Thus, these regions appear to consist primarily of flexible disorder since only the motif is conserved while the surrounding disordered region is under less selective constraint and is presumably important in facilitating the promiscuous binding required for signaling proteins.

Another class of proteins associated with promiscuous protein binding, chaperone proteins, is clearly enriched for constrained disorder. While the importance of disordered regions in the functioning of chaperones is well established (for example, [30,31]), the role played by disordered regions in chaperones is still the subject of active investigation [32]. There are a number of hypotheses regarding the roles of disorder in protein chaperones, including the idea that disordered chaperones may directly or indirectly stabilize client proteins due to their high hydrophilicity, or the notion that disordered chaperones may help in shielding unfolded proteins from interactions with other molecules, and the aforementioned entropy transfer hypothesis (see [32] for a comprehensive review). Our study suggests that, regardless of the precise function of the disordered regions in chaperones, it differs from the role that disorder plays in signaling proteins.

Finally, the other major category of recognition proteins, 'permanent binding', appears to, at least in part, be populated by regions of constrained disorder. This is supported by the enrichment for ribosomal proteins that are known to fold upon binding other ribosomal proteins and rRNA. Again, we suspect that cases where disordered regions fold permanently upon binding other molecules will be enriched for constrained disorder due to increased selective pressure required to maintain a stable bond.

Another classification scheme for disordered regions was put forth in [27] based on an unsupervised, data-driven partitioning of 145 disordered proteins, which identified three 'flavors' of disorder. The group of proteins described as 'flavor V' is highly enriched for ribosomal proteins and resembles the enrichments of constrained disorder defined here, while 'flavor S' was highly enriched for protein binding functions similar to regions of flexible disorder. However, these categories only weakly resemble the flexible and constrained disorder defined here as evidenced by their apparently distinct amino acid distributions (compare Figure 1 of [27] and Figure S25 in Additional file 1). These differences may stem from the fact that the previous classification scheme used an unsupervised algorithm and a limited set of proteins, and, most importantly, trained on whole proteins. In other words, it assumed that all disorder in one protein is of the same category, an assumption we are not making.

## Conclusions

In this work, we show that protein disorder can be partitioned into three biophysically and biologically distinct phenomena. The first two, flexible ('classic') and constrained disorder, capture different functional characteristics. On the other hand, non-conserved disorder does not seem to have functional roles. Our results have wide-ranging consequences for the prediction of disordered regions and for the functional interpretation of disordered regions in cellular networks. Future experimental work may confirm the distinct biophysical properties of constrained and flexible disorder we are predicting here. Importantly, our analysis framework allows for much more detailed functional interpretations of disordered regions. Finally, our new categories of disorder will help in the refinement of disorder prediction algorithms.

## Materials and methods

### Description of gene/protein level features and correlation analysis

Throughout this paper, correlations were done using Pearson's correlation coefficient [33] and calculated using Matlab's *corrcoef *function. Error bounds are the 95% confidence interval.

In the following section, we describe the data sets used throughout to characterize aspects of disorder. Several of these features were previously described in [9].

#### Genetic interaction degree

This was the same measure as the negative GI degree in [9]. Specifically, it is the number of negative interactions each array gene has, where negative interactions are defined as those that have a score ε < -0.08 and *P *< 0.05.

#### Protein disorder

Protein disorder was derived using the software Disopred2 [2]. We define structured proteins to be those with less than 10% disorder and disordered proteins to be those with greater than 30% disorder, following [4].

#### dN/dS Ratio

We computed the average dN/dS ratio for *S. cerevisiae *in comparison to the yeast species (*Saccharomyces paradoxus*, *Saccharomyces bayanus *and *Saccharomyces mikatae*). Sequences were subsequently aligned using MUSCLE [34] and dN/dS ratios were computed using PAML [35].

#### Expression level

The expression level of a gene as measured by the average number of mRNA copies of each transcript per cell were taken from [36].

#### Half-life

The half-life of a gene was the half-life of its mRNA measured in minutes and reported in [37].

#### Phenotypic capacitance

The phenotypic capacitance reflects the variability in a panel of phenotypes induced by deletion of non-essential genes and was used directly from the Levy and Siegal study [38].

#### Multi-functionality

This is simply the number of GO process annotations for each gene restricting to the functionally distinct set of GO terms described in [39].

#### Expression coherence score

This is the clustering coefficient calculated on the MEFIT [40] combined network where edges are genes with a score higher than 2 (approximately 95th percentile). Let *E*(N_i_,N_j_) be 1 if there is an edge between N_i _and N_j _and zero otherwise. The clustering coefficient for a gene G with n neighbors {N_i_} is:

#### Linear motifs

Linear motifs were found using Scansite [41] on the most stringent setting.

### Conserved disorder

#### Defining conservation of disorder and sequence residues from the yeast clade

Each of 5,025 orthologous groups across 23 species in the yeast clade [42] was multiple-aligned by MAFF [43] with default parameters. Amino acid conservation scores (A) of each position in each alignment was calculated and binned as follows:

where *a*_*i *_represents one of 20 different amino acid symbol indicator functions in an alignment position in *k*^*th *^protein sequence, and *N *stands for total number of protein sequences aligned.

For disorder conservation score (D), each alignment position was overlaid with the disorder symbol predicted by Dispred2 [2] with default parameters and its conservation was calculated and binned as follows:

where *d *represents the disorder indicator function in an alignment position in *k*^*th *^protein sequence.

We only considered proteins for which at least ten orthologs were available, and residue positions where at least five of those orthologs were aligned. All orthologous sequence- and disorder-overlaid alignments are displayed with the Jalview applet [44] and available at [45].

#### Structural conservation of disorder calculation

To calculate how disorder is structurally conserved despite changes at the amino acid level, we divided amino acids into a group associated with disorder and a group associated with structure (Table 1). This list was compiled based on [6] where disordered amino acids are charged and hydrophilic while structural amino acids are neutral and therefore hydrophobic. We considered all positions of orthologs of *S. cerevisiae *genes after alignment. If the amino acid was changed from *S. cerevisiae *to an ortholog, we recorded if it was changed towards the disordered set of amino acids or the structured set of amino acids. Then we compared the amino acids in regions of conserved disorder, regions of non-conserved disorder and the background (all positions).

**Table 1:** Description of the structural and disordered classes of amino acids

Structural amino acids	Disordered amino acids
Cysteine	Aspartic acid
Tryptophan	Methionine
Tyrosine	Lysine
Isoleucine	Arginine
Phenylalanine	Serine
Valine	Glutamine
Leucine	Proline
Histidine	Glutamic acid
Threonine	Alanine
Asparagine	Glycine

### A systematic classification of disorder

#### Definitions of constrained and flexible disorder

Conserved disorder: aligned positions that have D ≥ 5, that is, are disordered in more than 50% of aligned residues.

Flexible disorder: aligned positions that have D ≥ 5 and A < 5, that is, are disordered in greater than or equal to 50% of aligned residues but are conserved in less than 50% of aligned residues.

Constrained disorder: aligned positions that have D ≥ 5 and A ≥ 5, that is, are disordered in greater than or equal to 50% of aligned residues and conserved in greater than or equal to 50% of aligned residues.

Non-conserved disorder: aligned positions that have D < 5, that is, are disordered in *S. cerevisiae *but are disordered in less than 50% of aligned residues.

#### Distribution of residues in two conservation spaces: phosphorylation and linear motifs

Phosphorylation sites of *S. cerevisiae*, *Schizosaccharomyces pombe and Candida albicans *were obtained from [46] and a compilation of phosphosite datasets [46-51]. Linear motif sites are predicted by ScansSite2.0 on the *S. cerevisiae *data. Each distribution of feature-residue-odds-ratio (*O*^*feature*^, termed as relative density in the main text and Figure 5a) is calculated in a similar way as the hub-odds-ratio:

where *F*_*ij *_represents the number of feature residues (that is, phosphorylation site) with *i*^*th *^amino acid conservation score (A) and *j*^*th *^disorder conservation score (D) in whole proteins of *S. cerevisiae*, *S. pombe*, *and C. albicans *in case of phosphorylation sites or *S. cerevisiae *in case of linear motifs.

Each distribution of phosphorylation-site-odds-ratio (P) and linear-motif-odds-ratio (M) is displayed with levelplot function in lattice R package [52]. Partial correlations of *O*^*feature *^and A (or *O*^*feature *^and D) are statistically tested by pcor.test function [53], and plotted with residuals after controlling each other by linear regression.

#### Distribution of two conserved residues in hubs: GI, protein-protein interaction and structural interaction network

The 50th or 90th percentile hubs of GI and protein-protein interaction networks were defined as proteins with the degree greater than 50th or 90th percentile degree in the respective degree distributions. Singlish- and multi-interface hubs were defined as described in [20] with the structural interaction network recently updated with *i*Pfam corresponding to Pfam release 21.0 [54], 2,295 yeast Protein Data Bank files [55] and 82,650 physical interactions in Biogrid 2.06 [56].

### Function of flexible versus constrained disorder

#### GO enrichments

We found GO term enrichments for disorder type (flexible, constrained and non-conserved disorder) using the following method. The distribution of disorder type for each GO term was tested against the background distribution of that disorder type using the Wilcoxon rank sum test for *P*-value < 0.05, where the *P*-value was adjusted for multiple hypothesis testing using Benjamini-Hochberg false discovery correction. Terms enriched for either flexible or constrained disorder were only considered enriched if the distribution of ratios (Flexible/(Flexible + Constrained) or Constrained/(Flexible + Constrained), respectively) was significantly higher than the background for the term using a Rank sum test with *P *< 0.01. Thus, a term that was reported as enriched for flexible disorder was not also enriched for constrained disorder. Similarly, terms that initially were enriched for non-conserved disorder were tested to see if the ratio (Non-conserved disorder)/(Total disorder) was above the background of the term using a Rank sum test with *P *< 0.01. Enrichments for flexible and constrained disorder are contained in Additional file 2.

#### Domain analysis

To define domains, we used the *domains.tab *file downloaded from the Saccharomyces Genome Database on 4 April 2010, which contains the results of an InterProScan using each *S. cerevisiae *protein sequence to query for domains/motifs from several databases. The file consists of 40,737 domains mapped onto the yeast proteome. To restrict our analysis to structural domains, we only considered 11,801 domains mapped using three methods: superfamily (SCOP database, 4,943 domains), HMMPfam (Pfam database, 4,422 domains) and Gene3D (CATH database, 2,336 domains). For each of the 3,680 genes with mapped domains and alignments, every position in the sequence was associated with two conservation scores: conservation in disorder (D) and conservation in amino acids (A) obtained from the sequence alignments of the yeast clade (see above). For a given point in the conservation grid (A, D), we counted the residues that overlap with at least one domain and the residues that did not overlap with any domain. We then computed the log odds ratio of these counts.

#### Family analysis

For each domain, we computed the percentage of residues falling in each of the three categories: flexible disorder, constrained disorder, non-conserved disorder. We then compared the distributions of these percentages for all domains. We extracted the domains enriched in constrained disorder as opposed to flexible disorder by examining the ratio constrained/flexible (false discover rate (FDR)-adjusted Wilcoxon *P*-value < 0.05). The tests were performed with the function *wilcox.test *and the *P*-values were corrected for multiple testing with the function *p.adjust(method = 'FDR') *from the statistical programming environment R [52]. The results of these enrichments are contained in Additional file 2.

#### Enrichment map

Enrichment maps were created using Cytoscape [57] and the Enrichment Map plugin [58]. The edges represent the value of the overlap coefficient (size of the intersection of both GO terms/size of the small GO term) with a cutoff at 0.3.

## Abbreviations

GI: genetic interaction; GO: gene ontology; IDP: intrinsically disordered proteins.

## Authors' contributions

JB, CM and PK conceived the project. JB, SH, MM and TK designed and implemented the analysis. JB, SH, MM, MC, BA, CB, GB, CM and PK wrote the paper. CM and PK helped to develop the approach and supervised the research. All authors read and approved the final manuscript.

## Supplementary Material

Additional file 1 **Supplemental figures and tables**. This text file contains Figures S1 to S25 and Table S1 with their associated legends. Click here for file

Additional file 2 **Functional enrichment**. This file contains three tables: a table of GO terms (function, process and component) that are enriched for flexible and constrained disorder, a table of enrichments for domains in regions of constrained disorder and a table of enrichments for domains in regions of non-conserved disorder. Click here for file
