# Supplementary material for: Bringing order to protein disorder through comparative genomics and genetic interactions
Source: Genome Biol. 2011 Feb 16;12(2):R14. doi: 10.1186/gb-2011-12-2-r14 (PMC3188796; doi:10.1186/gb-2011-12-2-r14)
Supplement: Additional file 1 — Supplemental figures and tables. This text file contains Figures S1 to S25 and Table S1 with their associated legends. [file gb-2011-12-2-r14-S1.DOC]

**Bringing order to disorder: genomic analysis uncovers three distinct forms of protein disorder**

Jeremy Bellay^1*^, Sangjo Han^2,3*^, Magali Michaut^2,3*^, TaeHyung Kim^2,3^, Michael Costanzo^2,3^, Brenda J. Andrews^2,3,4^, Charles Boone^2,3,4^, Gary D. Bader^2,3,4,5^, Chad L. Myers^1^¶ and Philip M. Kim^2,3,4,5^¶

^1^Department of Computer Science and Engineering

University of Minnesota

200 Union Street SE

Minneapolis, MN 55455

USA

^2^The Donnelly Centre

^3^Banting and Best Department of Medical Research

^4^Department of Molecular Genetics

^5^Department of Computer Science

University of Toronto

160 College Street

Toronto, ON M5S 3E1

Canada

^*^These authors contributed equally to this work.

**¶** To whom correspondence should be addressed:

Tel: +1 416 946 3419; Fax: +1 416 978 8287;

Email: [cmyers@cs.umn.edu](mailto:cmyers@cs.umn.edu), [pi@kimlab.org](mailto:pi@kimlab.org)

# Supplemental Figures and Tables

**Figures**

# Figure S1: Disorder predicts protein-protein interactions on GI hubs

We calculated PPI degree by combining two AP/MS high-throughput studies [59,60], and one yeast two-hybrid study [61]. In figure S1, we plot the mean PPI degree of the GI hubs in the labeled percentile bins.


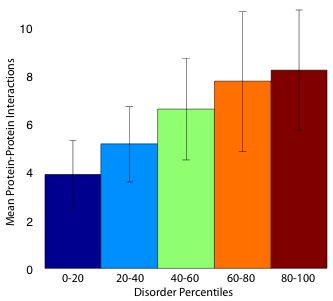


# Figure S2: Rate of change of disordered residues

We calculated how amino acids (AAs) change depending whether they are in regions of conserved disorder, non-conserved disorder or non-disorder. For each amino acid which varied between a protein in *S. cerevisiae* and the ortholog in another yeast species, we found the proportion that changed into an AA associated with disorder or one not associated with disorder (see Methods). As can be seen, AAs in conserved disordered regions are biased to change to other AAs that also support disorder, and are biased against AAs that are associated with structured regions. In non-disordered regions there is no distinction from random.


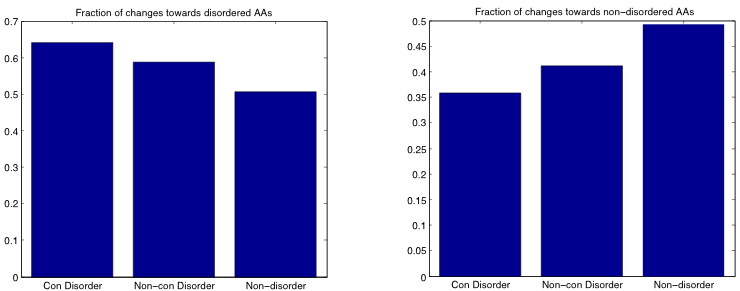


# Figure S3: Abundance of types of disorder

In the following pie chart we show the prevalence of the three classes of disordered residues across the *S. cerevisiae* genome.


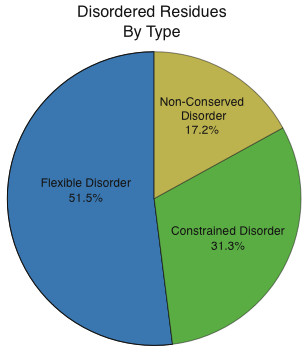


# Figure S4: Correlation on disorder types as defined by DisEMBL

We replicated figure 2B using the disorder predictor DisEMBL to classify residues as disordered instead of DISOPRED2. Residues that were either Coil, Hot loop or REM456 were considered to be disordered. Flexible, constrained and non-conserved disorder were defined as described in figure 2A. As can be seen below, using a different predictor does not change the trends between disorder type and the various gene/protein properties.


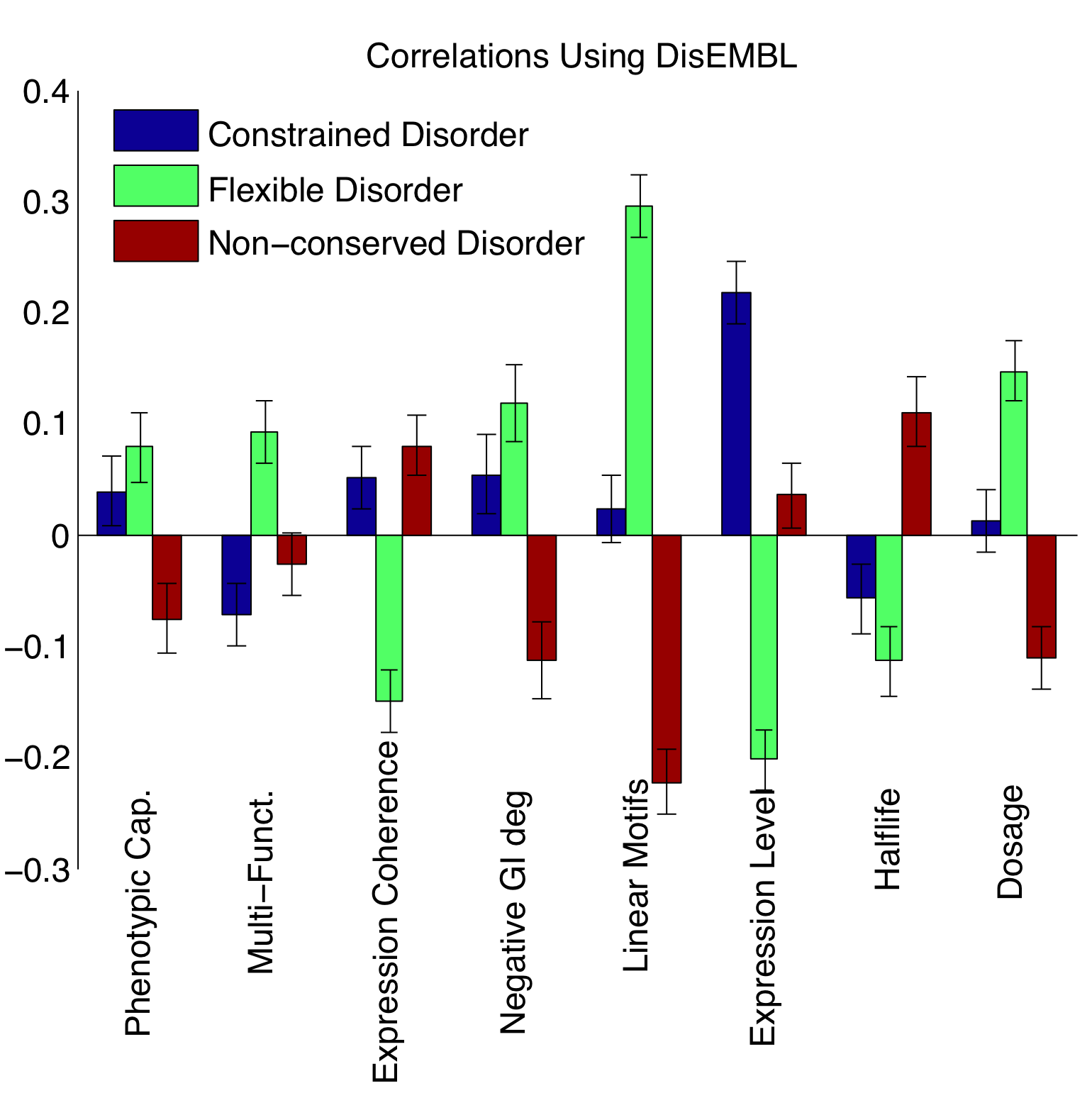


# Figure S5: Varying the amino acid conservation cutoff

We tested the stability of our results with respect to the cutoffs that determine what residues were classified as flexible, constrained, and non-conserved disorder. In the following figures, we show the correlations of Fig 2B when the amino acid conservation cutoff is varied between 6 (A) and 4 (B).

#
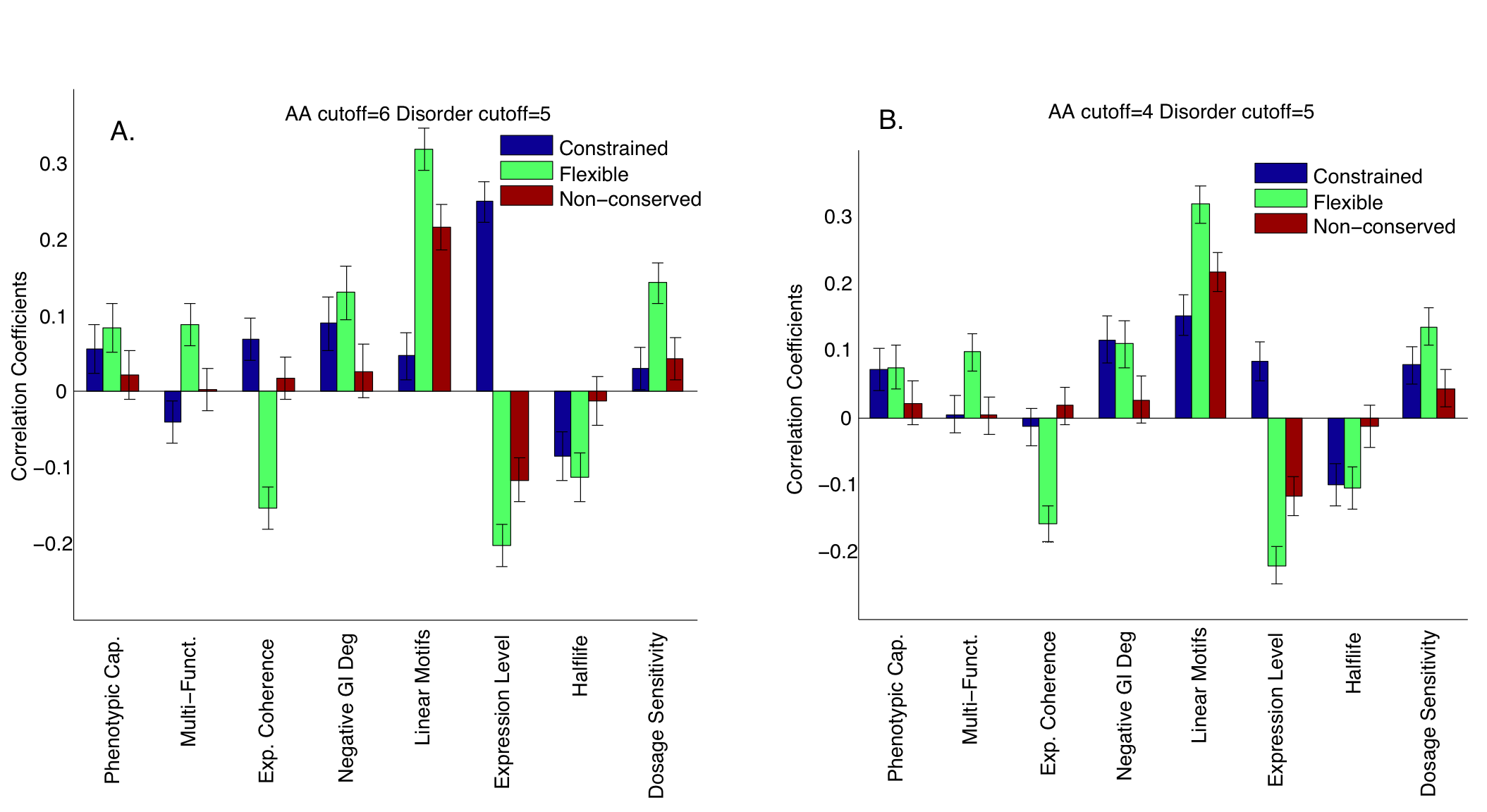


# Figure S6: Varying the disorder conservation cutoff

In the following figures we show the correlations of Fig 2B when the disorder conservation cutoff is varied between 6 (A) and 4 (B).

#
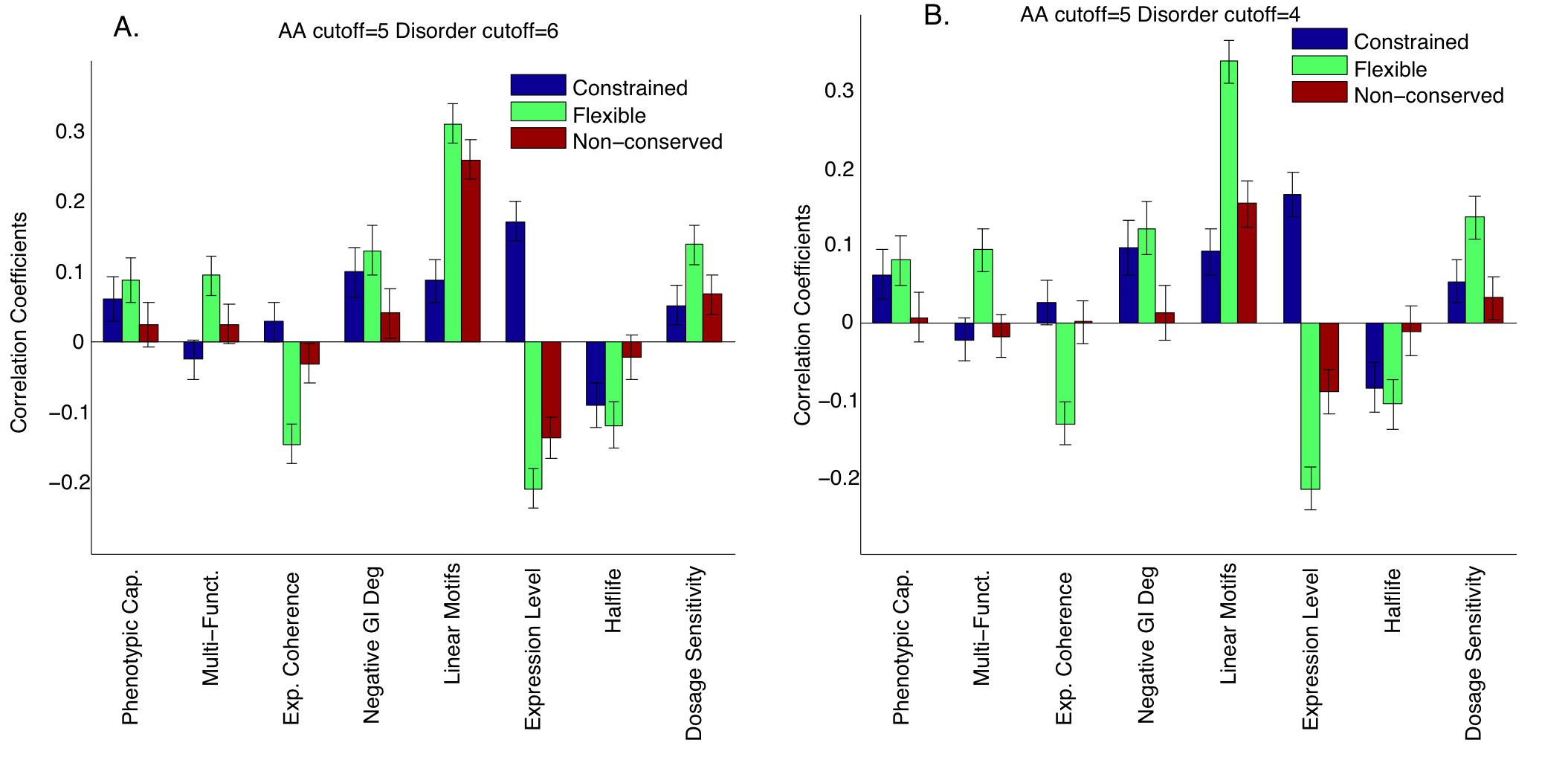


# Figure S7: Correlations without translation genes

We reproduce Fig 2B after removing all genes annotated to the GO process term “translation.” While the positive correlation between expression and constrained disorder becomes insignificant, the negative correlation between flexible disorder and expression remains.


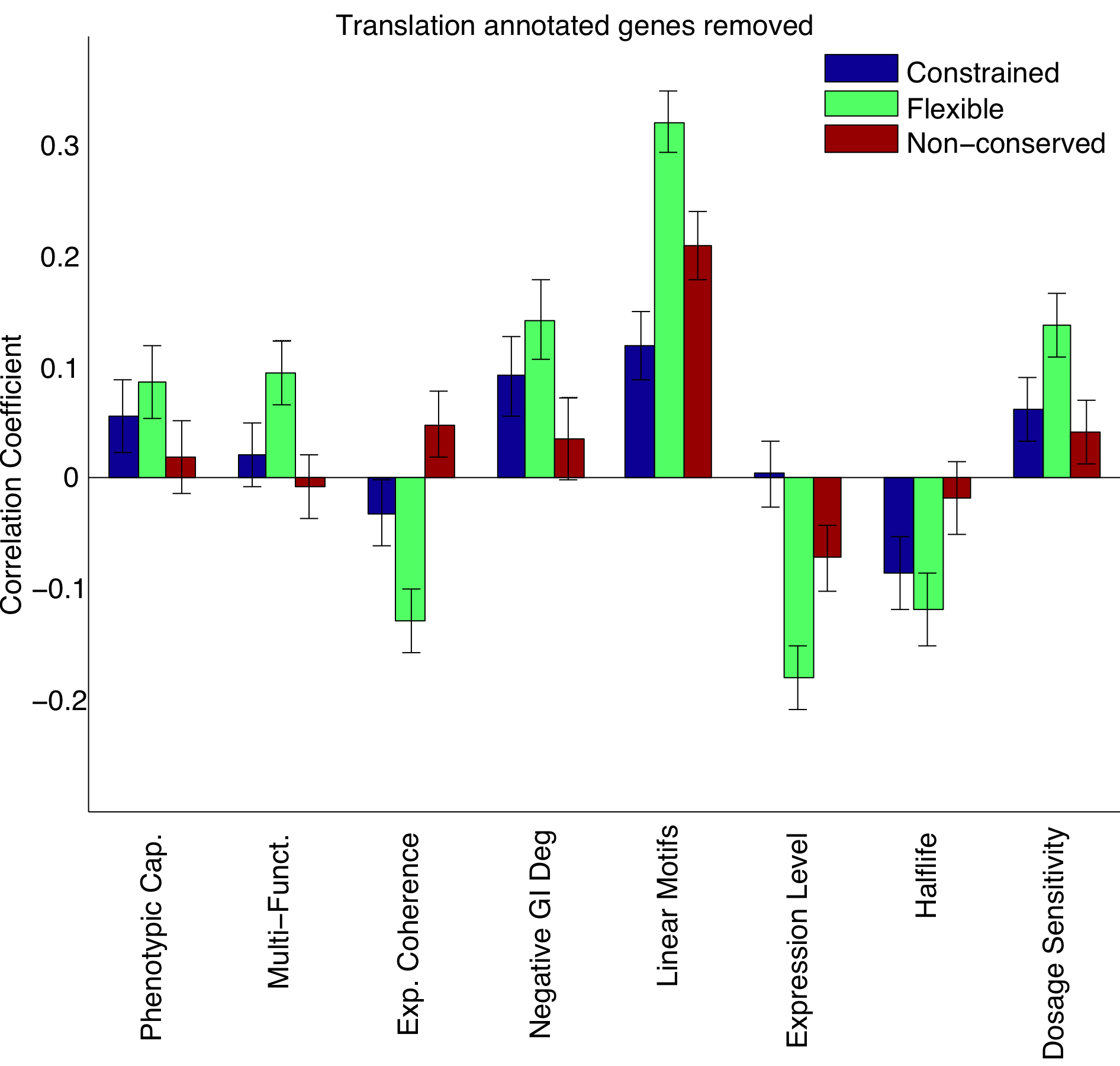


# Figure S8: Varying number of species used to calculate types of disorder

We randomly sampled 10 species from the complete set of 23 at random 20 times and defined the classes of disorder using the same rules described in Methods for each of the samplings. The following shows the mean correlation coefficients of the degree of disorder of each type with various functional and evolutionary properties across the 20 iterations, and the error bars are the standard error among those 20 iterations.

#
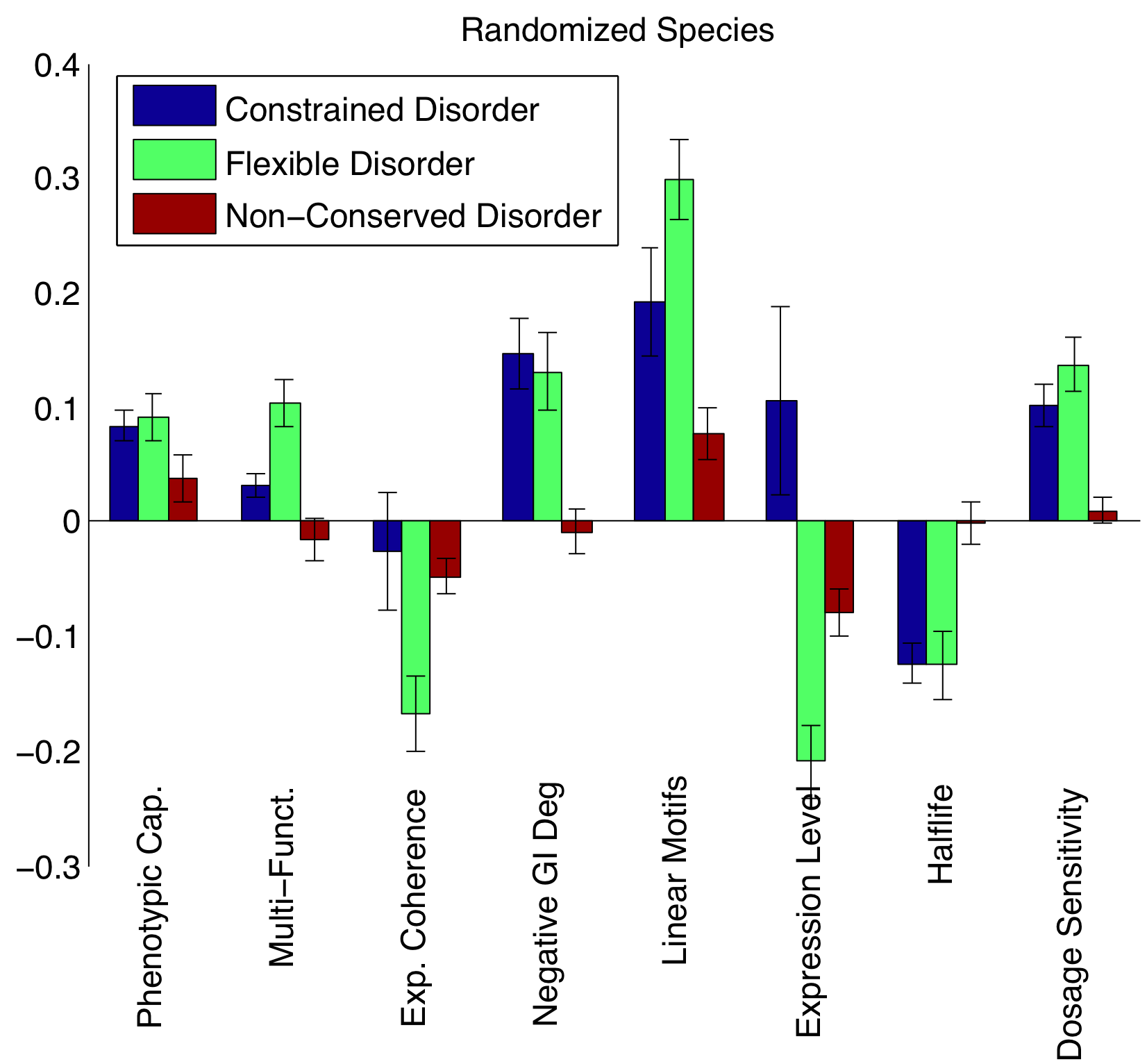


# Figure S9: The persistence of conserved disorder after simulated mutations

We introduced random mutations into 9,252 proteins of 478 randomly selected yeast ortholog groups. For each protein, we generated 157,286 randomly mutated versions at each of the following mutation frequencies: 0,2,4,6,8,10,15,20,25,30,40,50,60,70,80,90 and 100, for a total of 19,040 randomly mutated proteins. For each randomly mutated protein, we predicted disordered residues using Disopred2 as well as aligned them with MAFF. Subsequently, those predicted disorder residues were overlaid on multiple alignments of each ortholog group. Conserved, flexible, and constrained disorders were calculated as described in the Methods.

#
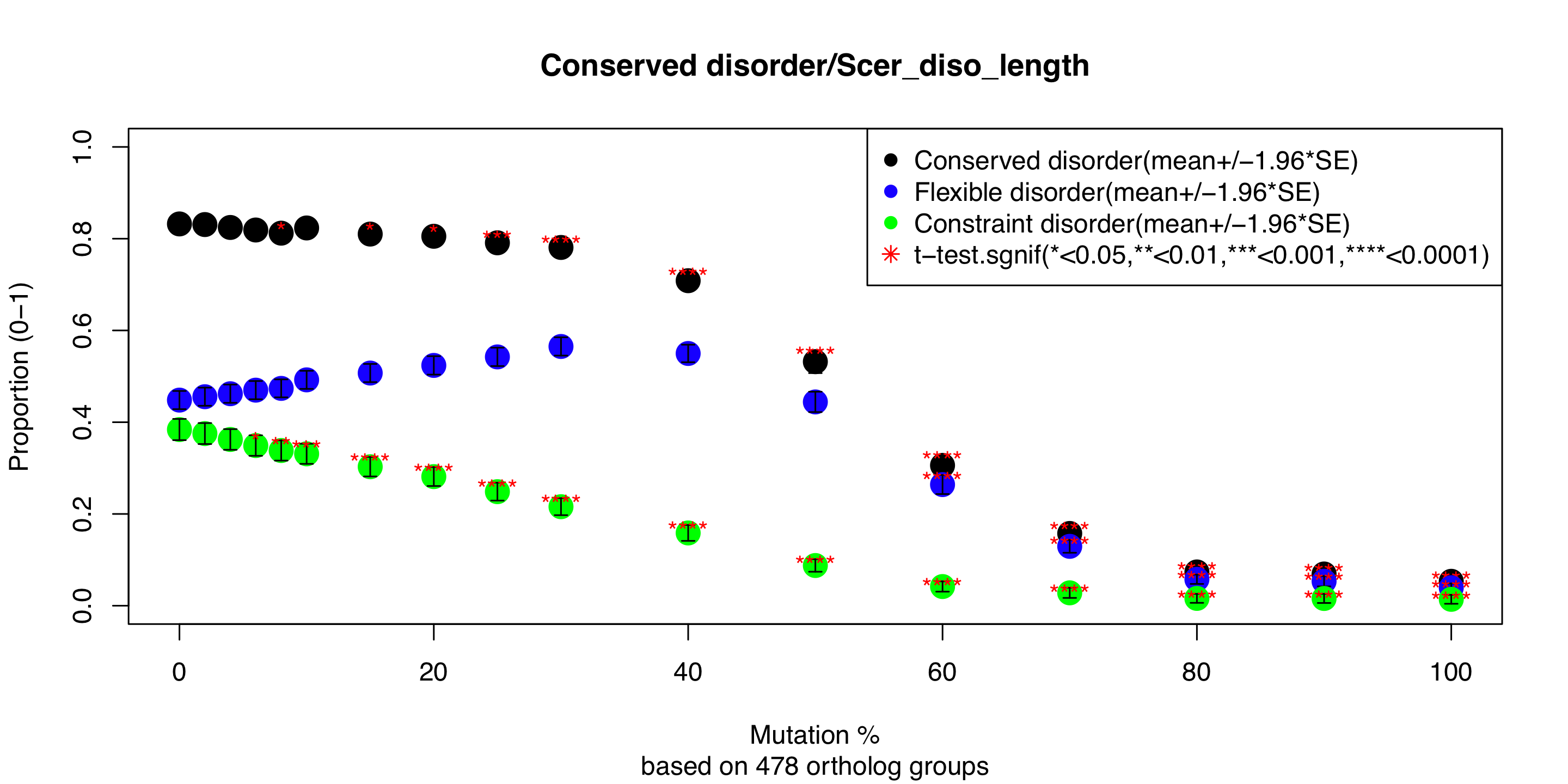


# Figure S10: Non-conserved disordered proteins have lower disorder scores

This figure shows the distribution of the disorder scores for two sets of proteins. Operationally we consider that a residue is disordered if its Disopred2 prediction score is higher than 0.05. For each protein in *S. cerevisiae*, we consider the prediction scores of all disordered residues and average them. We compare here the distributions of these averages for proteins with a lot of non-conserved disorder (> 50% of its residues) and few non-conserved disorder (<20% of its residues). These distributions show significantly different means (Wilcoxon test, p < 7E-5). Proteins with many non-conserved disordered residues tend to have lower prediction scores.


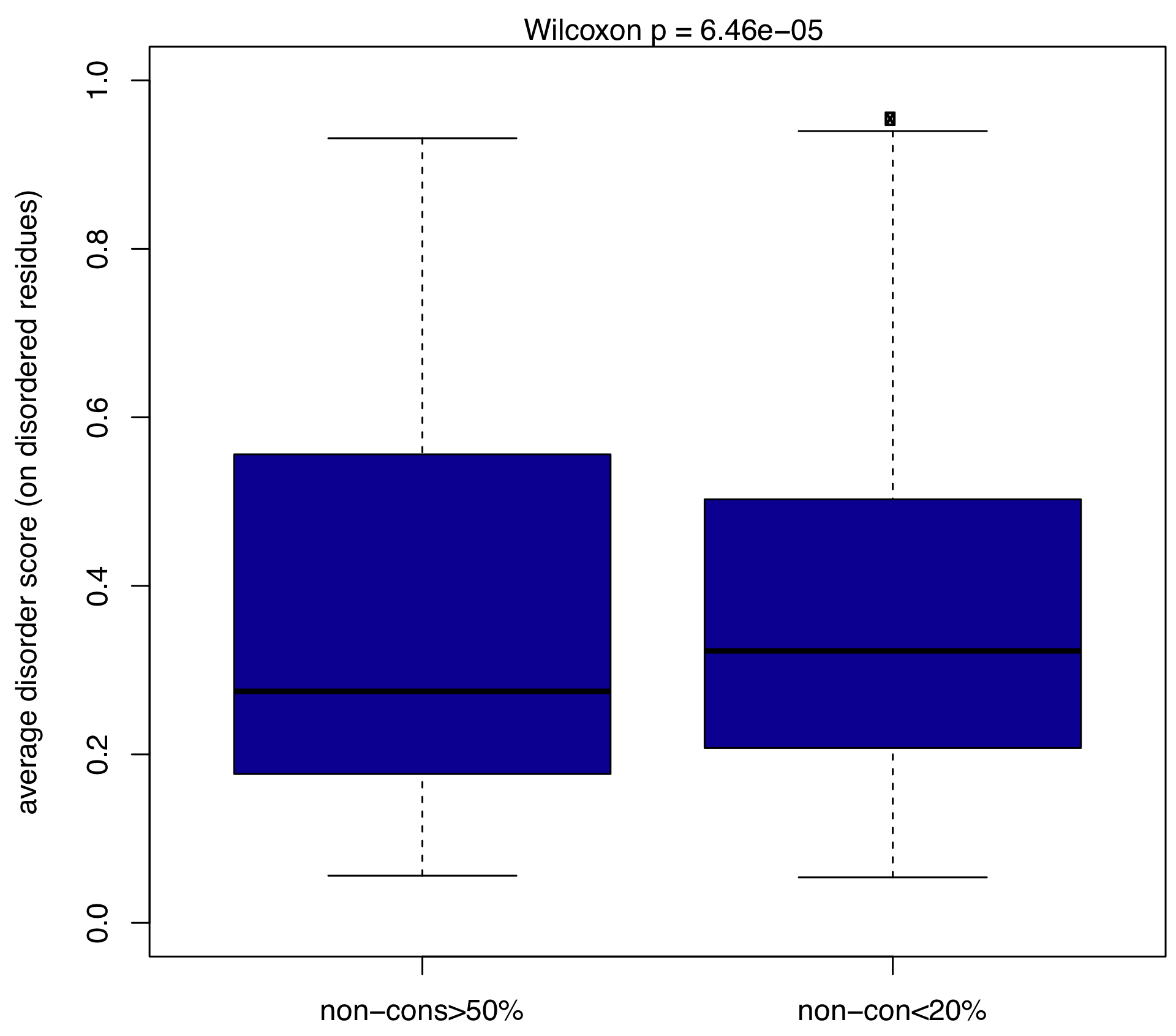


# Figure S11: Linear motif placement in conserved disorder

The occurrence of linear motifs shows an interaction with conservation of disorder (A). There is a significant partial correlation between disorder conservation and linear motif density when controlling for AA conservation (B), but there is no significant partial correlation between AA conservation and linear motif density when controlling for disorder conservation. (C).
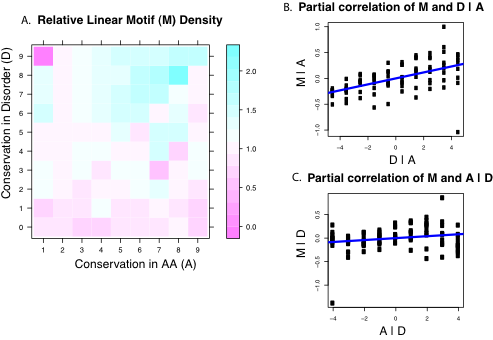


# Figure S12: Genetic interaction hubs are slightly enriched for conserved disorder

We found that GI hubs are enriched for conserved disorder (proportion test, p < 2 x 10^-30^). This enrichment appears to be dominated by an enrichment for constrained disorder (p < =5 x 10^-69^) rather than flexible disorder (p < 0.02). We plot the proportions of types of disorder among hubs and non-hubs in the following figure.


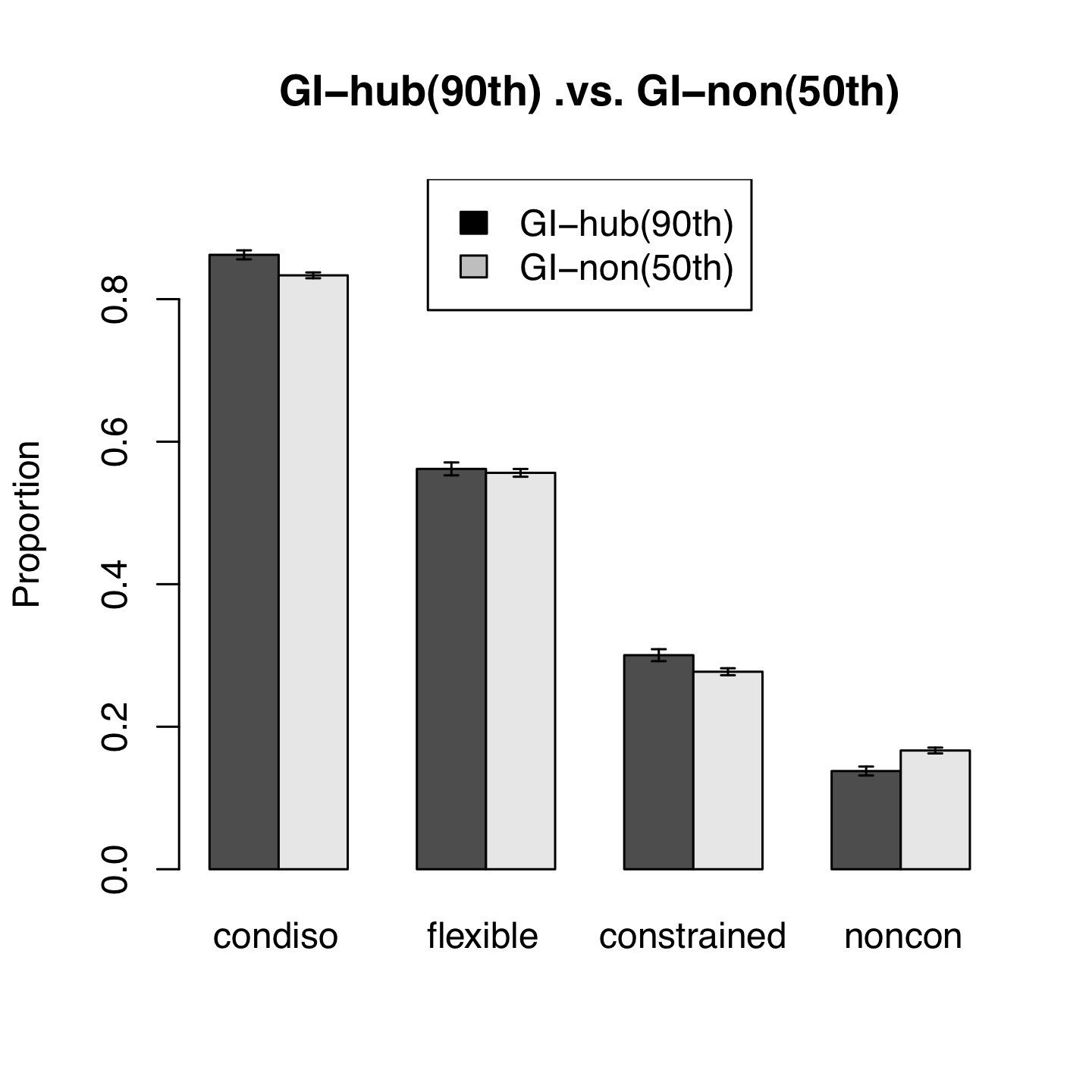


# Figure S13: The relationship between disorder and singlish/multi-interface hubs.

It was previously reported that singlish hubs were enriched for disorder while multi-interface hubs were not [62]. We find that even within the set of disordered proteins, GI hubs are extremely enriched for SI hubs (A). Moreover, as we report in this paper, the SI hubs are in particular enriched for flexible disorder (B), while seemingly all disorder present in multi-interface hubs is constrained disorder (C).

For comparison of distribution of two types of conserved residues in each hub definition, the hub-odds-ratio (*O^hub^*) is calculated as follows:

$$O_{ij}^{hub}=\frac{S_{ij}}{S_{N}}\frac{T_{N}}{T_{ij}}$$

where ***S_ij_*** represents the number of residues with ***i^th^*** amino acid conservation score (A) and ***j^th^*** disorder conservation score (D) in a given subset of proteins (i.e. hub proteins) and ***T_ij_*** stands for the number of residues with ***i^th^*** A and ***j^th^*** D in whole proteins of *S. cerevisiae*. ***S_N_*** and ***T_N_*** mean the number of total residues of proteins considered in a given subset and whole, respectively. Each odds-ratio distribution of GI-, PPI-, SIN-, and non-hubs is displayed with levelplot function in lattice R package [52].

#
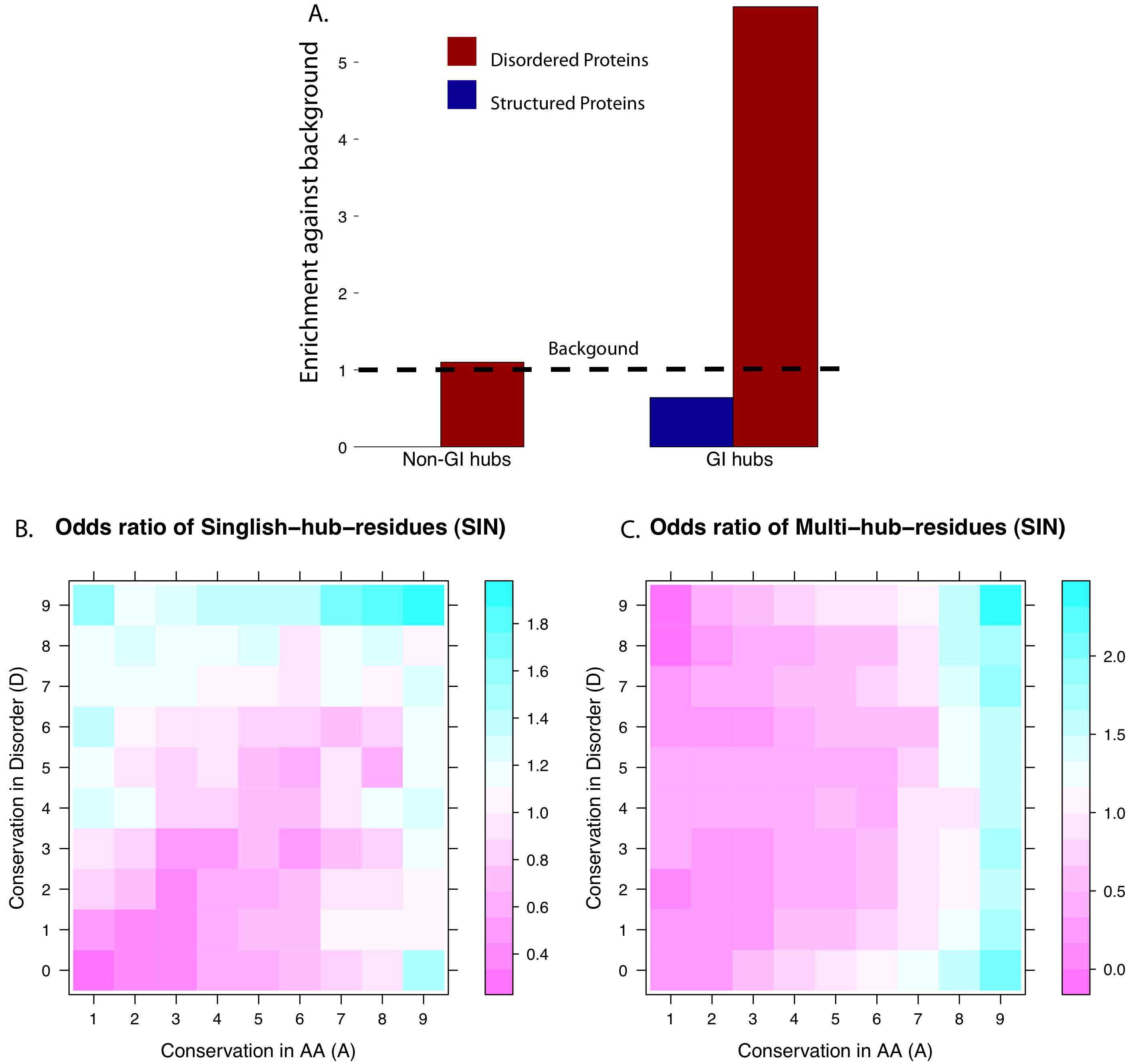


# Figure S14: Enrichment of domains in regions of flexible and constrained disorder

The percent of domains that exhibit flexible or constrained disorder. It is important to note that the background rate is 60% so both regions of flexible and constrained disorder are under-enriched for domains.


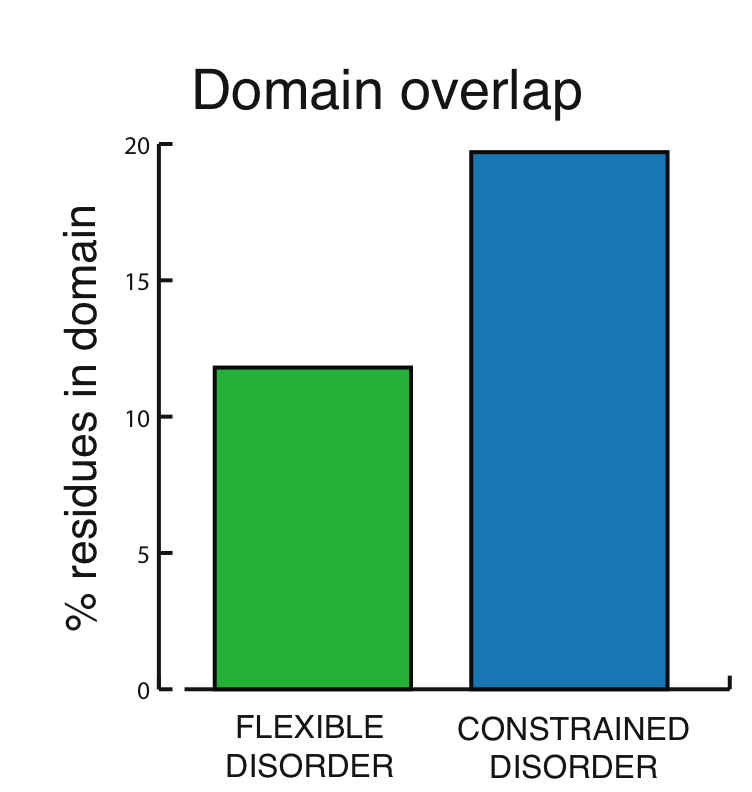


# Figure S15: Occurrence of domain in disordered regions

Neither flexible nor constrained disorder is enriched for domains with respect to the background rate. However, regions constrained disorder is significantly enriched for domains in comparison to the regions of flexible disorder.


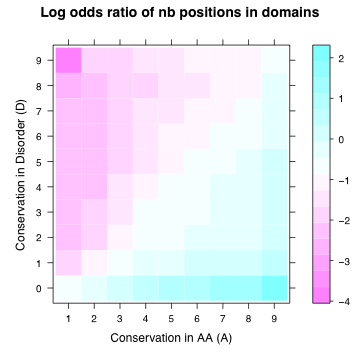


# Figure S16: An example of flexible disorder: Sky1

This example shows a region (AA 712-737) of flexible disorder in the serine-arginine protein kinase Sky1 (YMR216C). The left part shows the sequence and disorder conservation among the multiple alignments in the yeast clade. The disorder is conserved (red barplot), whereas the residues are not (blue barplot). The structure of the protein is shown on the right part of the figure with the specific region highlighted (in pink). This region is situated at the end of the kinase and consists of a C-terminal disordered loop, which interacts with the activation loop of the kinase.


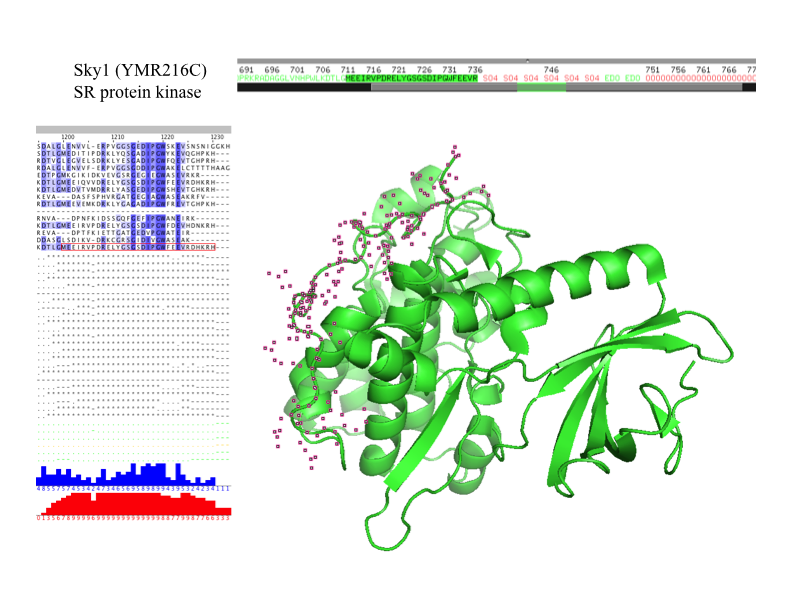


# Figure S17: An example of flexible disorder: Bur1

This figure shows the region of flexible disorder in BUR1 corresponding to the Sky1 loop. Moreover, this region is enriched for phosphosites and linear motifs.


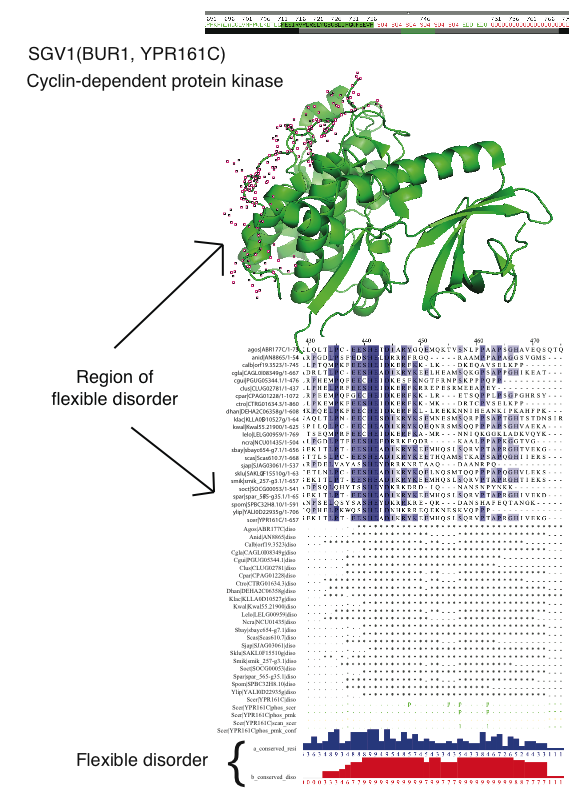


# Figure S18: An example of constrained disorder: Rpl5

This figure shows the C-terminal end of Rpl5 which was found by [23] to exhibit a disorder-to-order transition upon the binding of 5S rRNA.


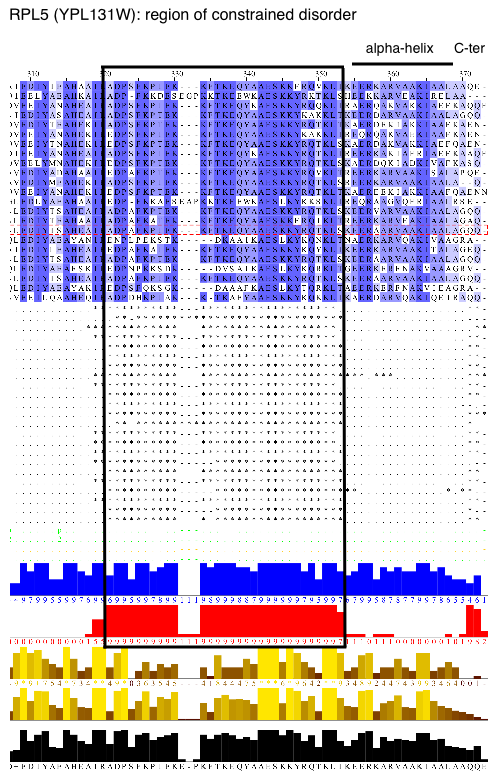


# Figure S19: An example of constrained disorder: HSC82.

The region 590-600 of constrained disorder is localized at the inner surface of the barrel-shaped heat shock protein.


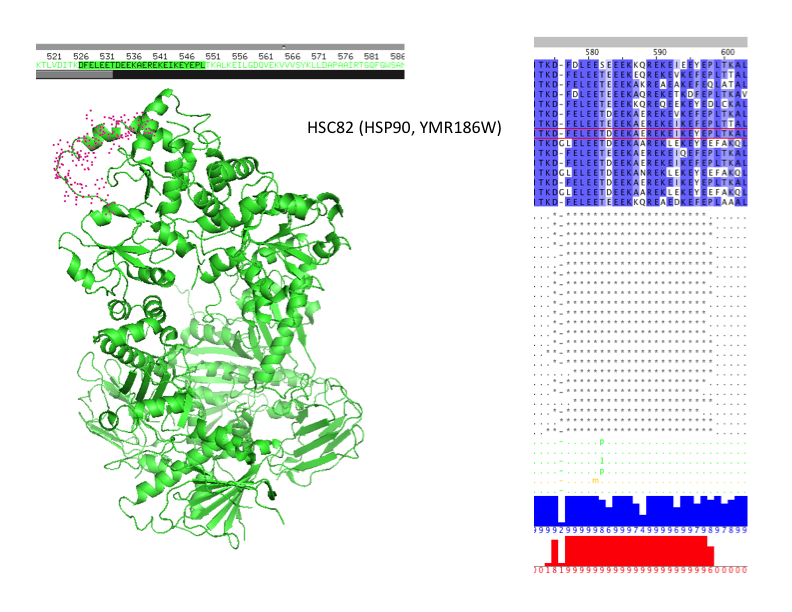


**Figure S20: Enrichment maps for flexible disorder:**

Each node is a GO term labeled by the name of the term and related GO terms are linked based on gene overlap (see Methods). The thicker the edge, the higher the overlap. The size of the nodes represents the size of the GO term (number of genes).

**
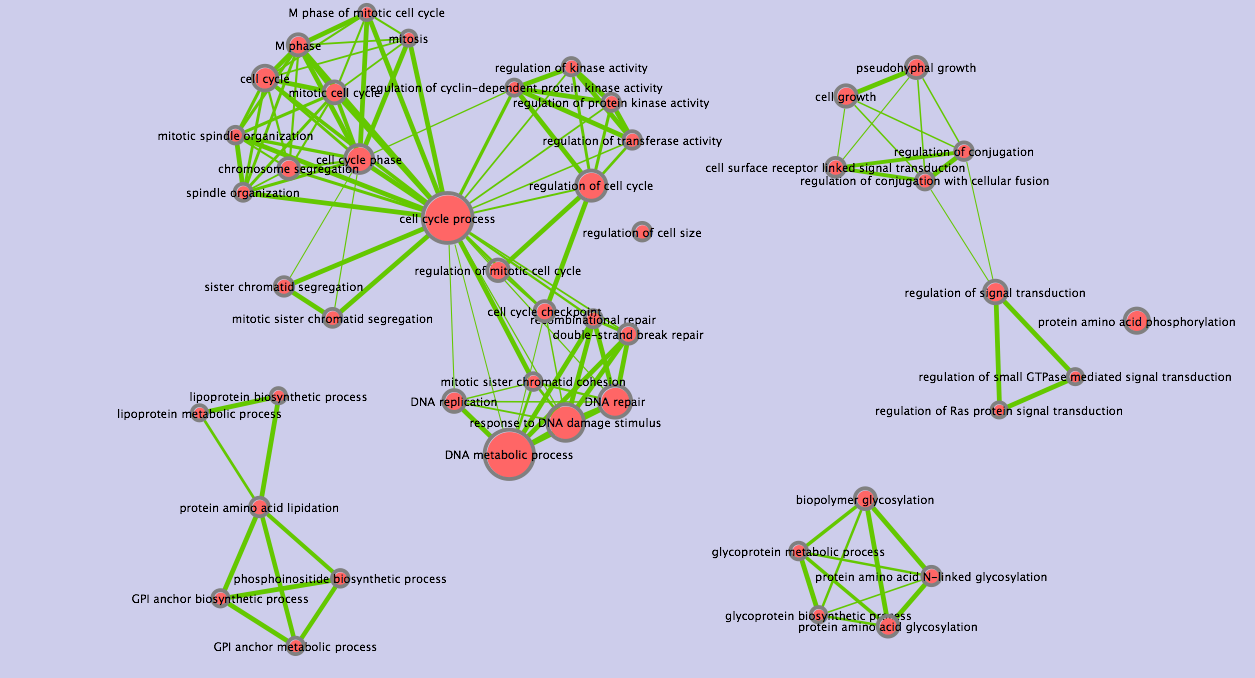
**

**Figure S21: Enrichment map for constrained disorder:**

Each node is a GO term labeled by the name of the term and related GO terms are linked based on gene overlap (see Methods). The thicker the edge, the higher the overlap. The size of the nodes represents the size of the GO term (number of genes).

**
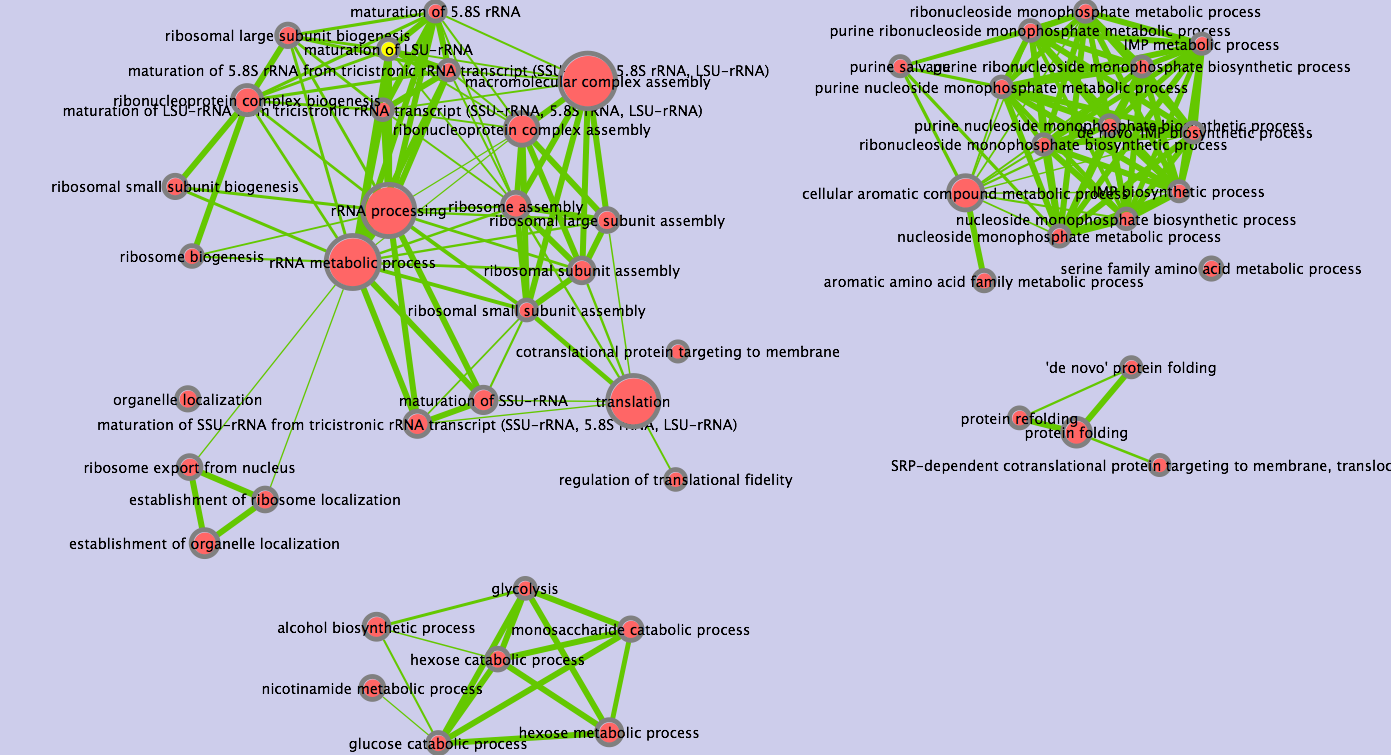
**

**Figure S22: Enrichment map for non-conserved disorder:**

Each node is a GO term labeled by the name of the term, and related GO terms are linked based on gene overlap (see Methods). The thicker the edge, the higher the overlap. The size of the nodes represents the size of the GO term (number of genes).

Many processes that enriched in non conserved disorder are related to transposition and DNA recombination. This is largely driven by transposons, in particular genes associated with the Ty1. Many of these *S. cerevisiae* genes start with Ty1-A and Ty1-B domains which are highly disordered and mainly consist of non conserved disorder residues (Fig S15). Ty1 elements are often known to be present but non-functional in yeast genomes, which may explain the lack of selective pressure on these disorder regions.

**
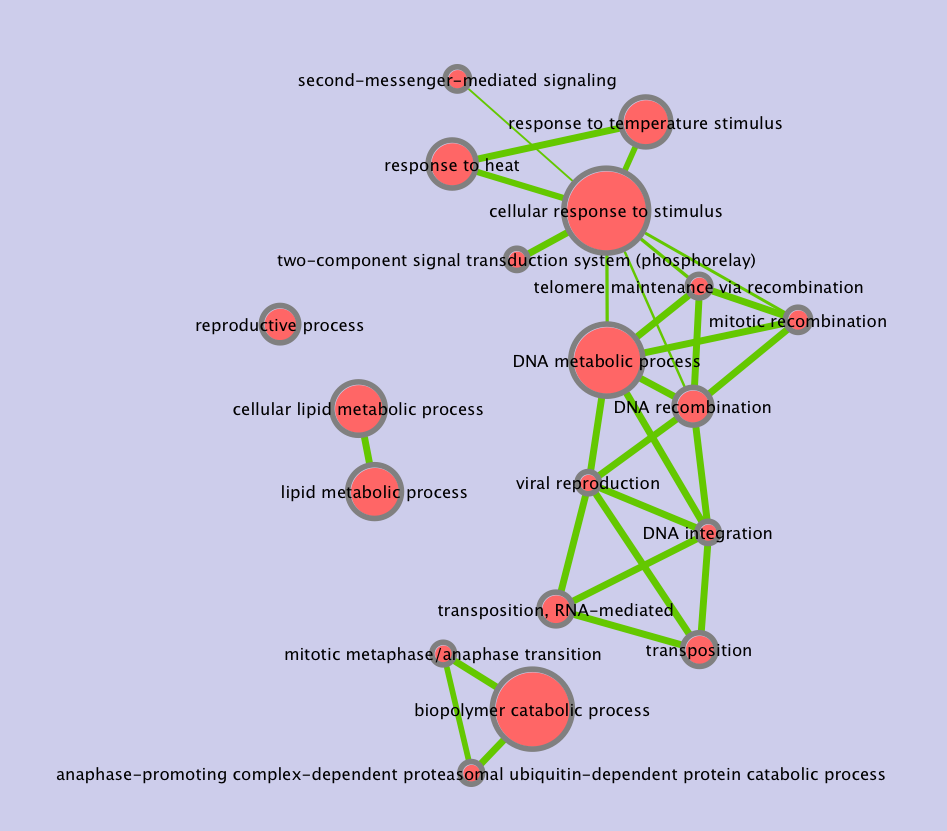
**

**Figure S23: An example of non-conserved disorder in a Ty1 gene.**

The Ty1 gene YHR214B-C contains the 2 domains Ty1-A and Ty1-B which consist of non conserved disorder regions.

**
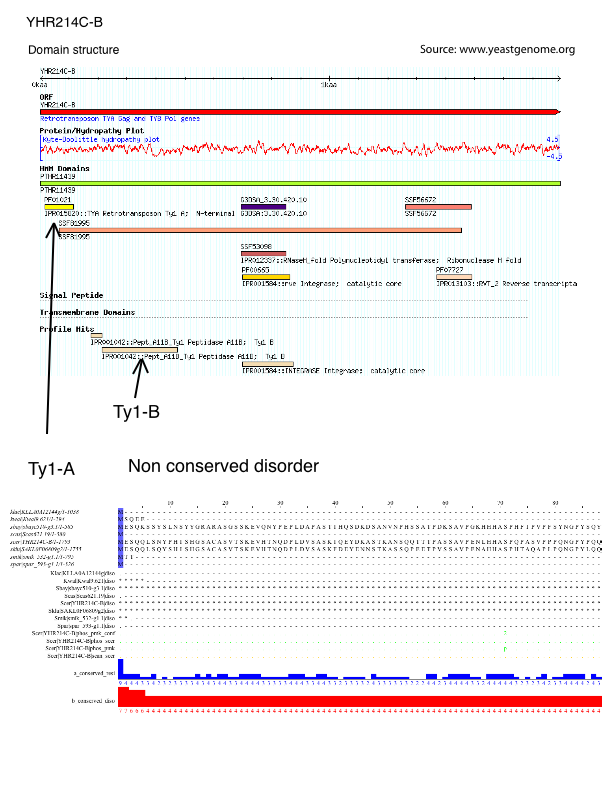
**

**Figure S24: Divisions of disorder from [26].**

A categorization of disordered proteins as proposed by Tompa in [26]. The proposed relationships between these divisions and the three classes disorder proposed here are indicated by color.


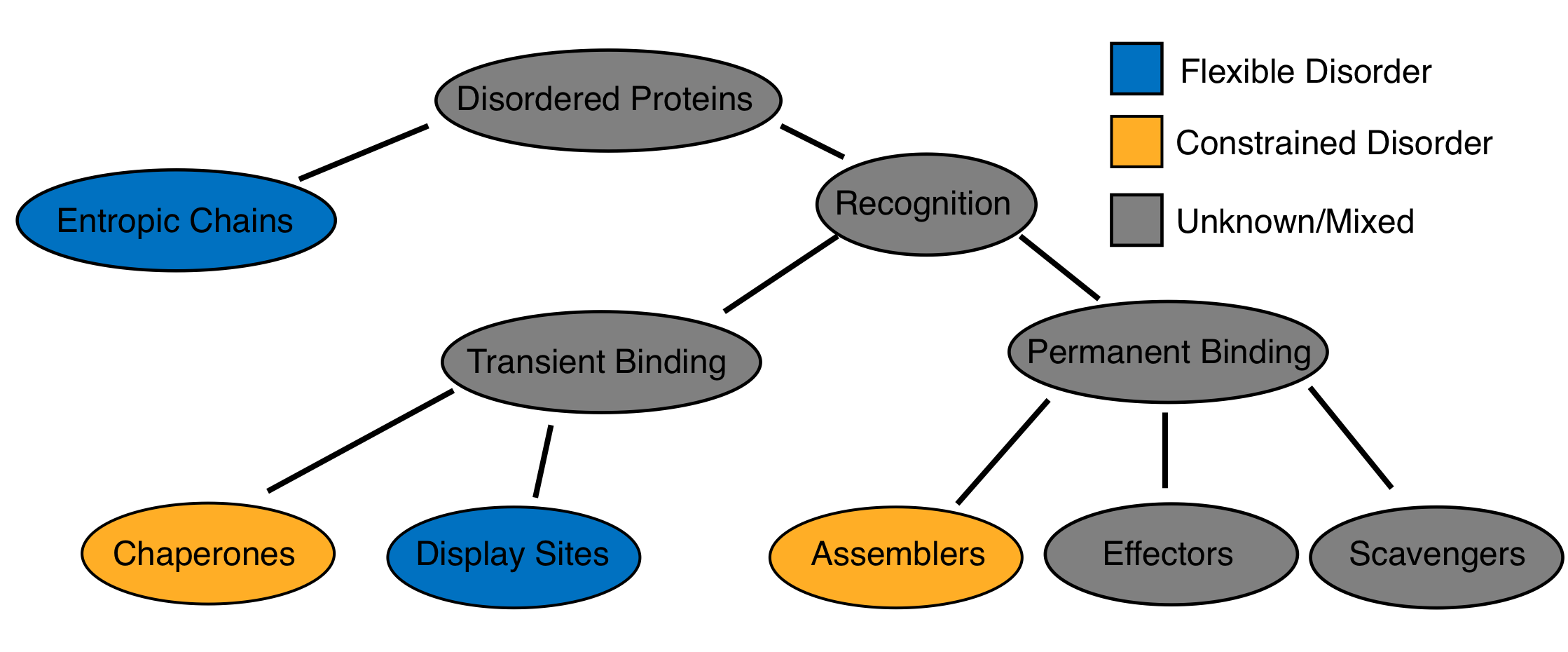


**Figure S25: Frequency of amino acids in the three types of disorder**

We investigated how frequently each amino acid was associated with each type of disorder. The following figure plots the log of the ratio of the frequency of a given amino acid in a type of disorder to the frequency of that amino acid in structured domains.

**
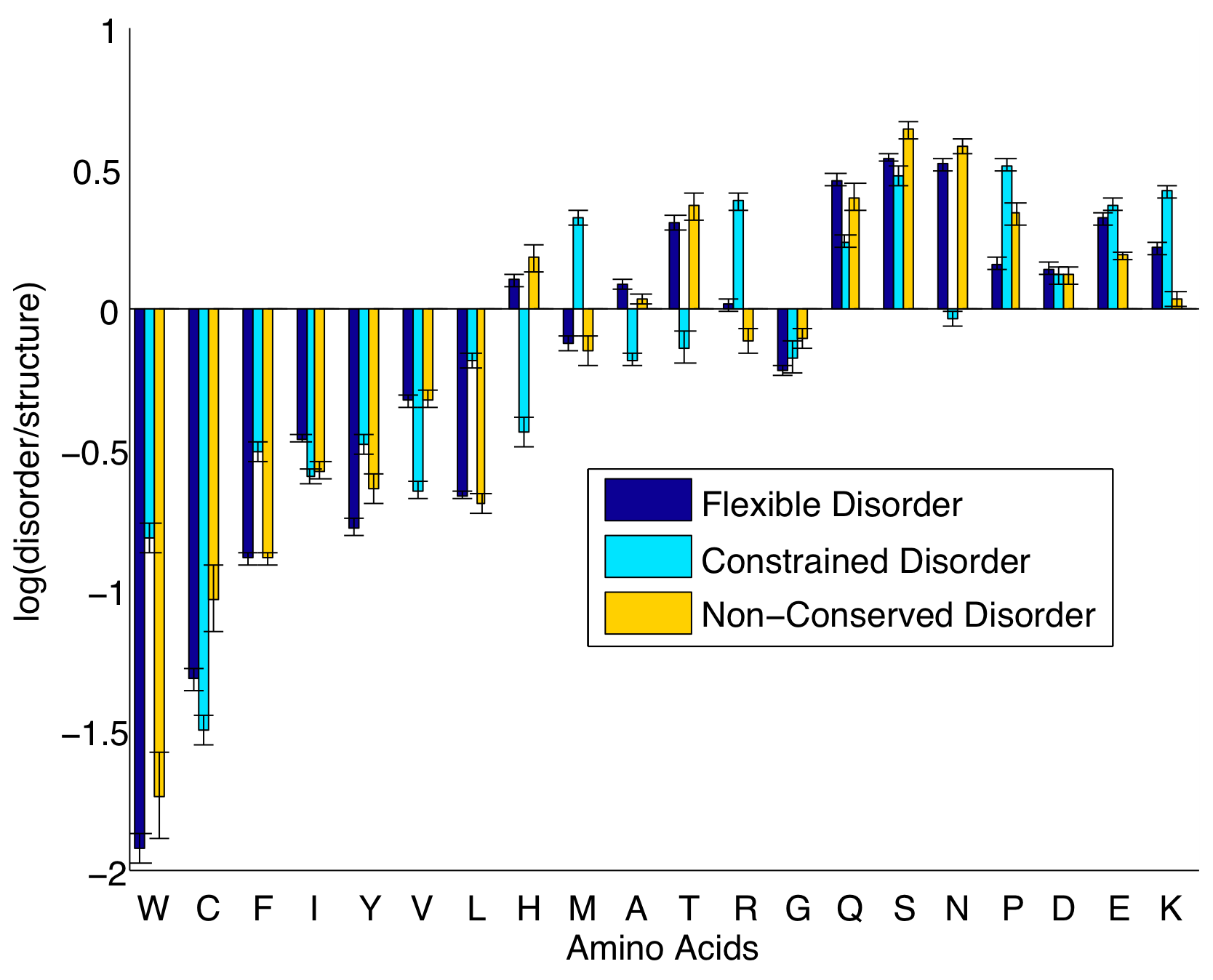
**

**Tables**

# Table S1: Correlation and T-test of Coefficient of variation and GI degree

To measure variation of percentages of disorder regions on proteins in an orthologous group, we used Coefficient of variation (CV) which is the standard deviation divided by the mean. In the same way, the variation of the number of disordered segments on proteins in an orthologous group is measured. First variation is defined as ‘orthoDiso.cv’, and second one as ‘orthoSeg.cv’. Then, correlations of these two variations and GI degree are investigated (Table S1). Both of orthDiso.cv and orthSeg.cv are negatively correlated with the GI degree. In the same line, differences of each variation are t-tested between GI-hub protein-containing groups and non- hub groups. Again, both of them are significantly lower in GI-hub groups compared with non-hub groups. It indicates that the variations of percentages or segment numbers in disordered regions of GI-hub proteins are smaller than those of non-hub proteins. In other words, disordered regions of GI-hub proteins seem to be more conserved than those of non-hub proteins.

|  | Pearson’s correlation test with GI degree | Student T-test between non-hub vs. GI-hub |
| --- | --- | --- |
| orthDiso.cv^1^ | -0.11  (P < 2 x 10^-9^) | 0.32 vs. 0.26  (P < 4 x 10^-12^) |
| orthSeg.cv^2^ | -0.06 (P < 0.001) | 0.323 vs 0.298  (P < 3x 10^-6^) |

^1^orthDiso.cv stands for coefficient of variation of % disorder of proteins in an orthologous group across 23 species. ^2^orthSeg.cv stands for coefficient of variation of the number of disordered segments of proteins in an orthologous group across 23 species.
